# Network communication models improve the behavioral and functional predictive utility of the human structural connectome

**DOI:** 10.1162/netn_a_00161

**Published:** 2020-11-01

**Authors:** Caio Seguin, Ye Tian, Andrew Zalesky

**Affiliations:** Melbourne Neuropsychiatry Centre, University of Melbourne and Melbourne Health, Melbourne, Australia; Melbourne Neuropsychiatry Centre, University of Melbourne and Melbourne Health, Melbourne, Australia; Melbourne Neuropsychiatry Centre, University of Melbourne and Melbourne Health, Melbourne, Australia; Department of Biomedical Engineering, Melbourne School of Engineering, University of Melbourne, Melbourne, Australia

**Keywords:** Brain network communication models, Neural signaling, Network neuroscience, Connectomics, Behavioral prediction, Structure-function coupling

## Abstract

The connectome provides the structural substrate facilitating communication between brain regions. We aimed to establish whether accounting for polysynaptic communication in structural connectomes would improve prediction of interindividual variation in behavior as well as increase structure-function coupling strength. Connectomes were mapped for 889 healthy adults participating in the Human Connectome Project. To account for polysynaptic signaling, connectomes were transformed into communication matrices for each of 15 different network communication models. Communication matrices were (a) used to perform predictions of five data-driven behavioral dimensions and (b) correlated to resting-state functional connectivity (FC). While FC was the most accurate predictor of behavior, communication models, in particular communicability and navigation, improved the performance of structural connectomes. Communication also strengthened structure-function coupling, with the navigation and shortest paths models leading to 35–65% increases in association strength with FC. We combined behavioral and functional results into a single ranking that provides insight into which communication models may more faithfully recapitulate underlying neural signaling patterns. Comparing results across multiple connectome mapping pipelines suggested that modeling polysynaptic communication is particularly beneficial in sparse high-resolution connectomes. We conclude that network communication models can augment the functional and behavioral predictive utility of the human structural connectome.

## Author Summary

Brain network communication models aim to describe the patterns of large-scale neural signaling that facilitate functional interactions between brain regions. While information can be directly communicated between anatomically connected regions, signaling between disconnected areas must occur via a sequence of intermediate regions. We investigated a number of candidate models of connectome communication and found that they improved structure-function coupling and the extent to which structural connectomes can predict interindividual variation in behavior. Comparing the behavioral and functional predictive utility of different models provided initial insight into which conceptualizations of network communication may more faithfully recapitulate biological neural signaling. Our results suggest network communication models as a promising avenue to unite our understanding of brain structure, brain function, and human behavior.

## INTRODUCTION

The structural connectome is a complex network that describes anatomical connections between neural elements (Bassett & Sporns, 2017; Fornito, Zalesky, & Bullmore, 2016). At the macroscale of magnetic resonance imaging (MRI), the human connectome delineates how gray matter regions are interlinked by white matter projections (Hagmann et al., 2008; Sporns, Tononi, & Kötter, 2005). Numerous studies have demonstrated that the macroscale human connectome is characterized by several nonrandom topological properties, including a small-world and modular architecture (Bassett & Bullmore, 2006; Sporns & Betzel, 2016), heterogeneous degree distribution (Bullmore & Sporns, 2009), and a core of densely connected hubs (van den Heuvel & Sporns, 2011). This complex anatomical scaffold both facilitates and constrains neural signaling between brain regions. While region pairs that share a connection in the structural connectome may communicate directly, polysynaptic paths comprising two or more connections are required to establish communication between anatomically unconnected regions. Understanding the dynamics of polysynaptic communication in large-scale brain networks is a key open challenge in neuroscience (Avena-Koenigsberger, Mišić, & Sporns, 2018).

Several network communication models have been proposed to describe large-scale neural signaling, ranging from naive random walk processes to optimal routing via shortest paths (Avena-Koenigsberger et al., 2019). By considering polysynaptic paths, these models capture communication between both connected and unconnected nodes, thus enabling a high-order structural description of interactions among every pair of regions in the connectome (Suárez, Markello, Betzel, & Mišić, 2020). Recent studies report that network communication models can improve the strength of coupling between structural and functional connectivity in the human connectome (Goñi et al., 2014), explain established patterns of cortical lateralization (Mišić et al., 2018), and infer the directionality of effective connectivity from structural connectomes (Seguin, Razi, & Zalesky, 2019). These efforts provide evidence that network communication models capture meaningful aspects of brain functioning and dynamics. However, the extent to which different models contribute to our understanding of neural signaling remains unknown.

Here, we aimed to systematically investigate the utility of a range of candidate models of network communication. First, we sought to determine whether modeling polysynaptic (multihop) communication in structural brain networks would (a) improve the prediction of interindividual variation in behavior, compared with predictions based on direct structural connections alone; and (b) improve the strength of structure-function coupling. Second, we aimed to establish a ranking of communication models with respect to their predictive utility, with the goal of determining which models may more faithfully capture biological signaling patterns related to behavior and FC.

We considered five previously proposed network communication measures: (a) shortest paths (Kaiser & Hilgetag, 2006; Latora & Marchiori, 2001), (b) navigation (Boguña, Krioukov, & Claffy, 2009; Seguin, van den Heuvel, & Zalesky, 2018), (c) diffusion (Goñi et al., 2013), (d) search information (Goñi et al., 2014; Rosvall, Grönlund, Minnhagen, & Sneppen, 2005), and (v) communicability (Andreotti et al., 2014; Crofts & Higham, 2009; Estrada & Hatano, 2008). Collectively, these models cover a widerange of neural signaling conceptualizations. Shortest paths and navigation deterministically route information using centralized and decentralized strategies, respectively. In contrast, diffusion and search information model communication from the stochastic perspective of random walk processes. Finally, communicability implements a broadcasting model of signaling, in which signals are simultaneously propagated along multiple network fronts. While all these candidate models have been investigated in the human connectome, which particular models provide the most parsimonious representation of large-scale neural signaling remains unclear.

Using diffusion-weighted MRI and tractography, we mapped structural connectivity (SC) matrices for 889 healthy adults participating in the Human Connectome Project (HCP; Van Essen et al., 2013). Each individual’s SC matrix was then transformed into a communication matrix, which represented the efficiency of communication between each pair of regions under a particular candidate model of network communication. For each model, communication matrices were fed to statistical techniques to perform out-of-sample prediction of individual variation in five behavioral dimensions (Tian, Margulies, Breakspear, & Zalesky, 2020), and also correlated with FC matrices mapped using resting-state functional MRI. This enabled a systematic ranking of network communication models in terms of behavior prediction and structure-function coupling. While these criteria do not constitute direct biological validation of signaling strategies, we hypothesize that the higher the predictive utility of a communication model, the more likely it is to parsimoniously recapitulate the signaling mechanisms of the human brain.

## RESULTS

### Brain Network Communication Matrices

Structural connectomes were mapped using white matter tractography applied to diffusion MRI data acquired for 889 healthy adults participating in the Human Connectome Project (Van Essen et al., 2013; See the Methods section). We focus on reporting results for connectomes comprising *N* = 360 cortical regions (Glasser et al., 2016) that were thresholded to eliminate potentially spurious connections (Zalesky et al., 2016). Results for alternative cortical parcellations and connection density thresholds are reported in the Supporting Information.

**Figure 1.**
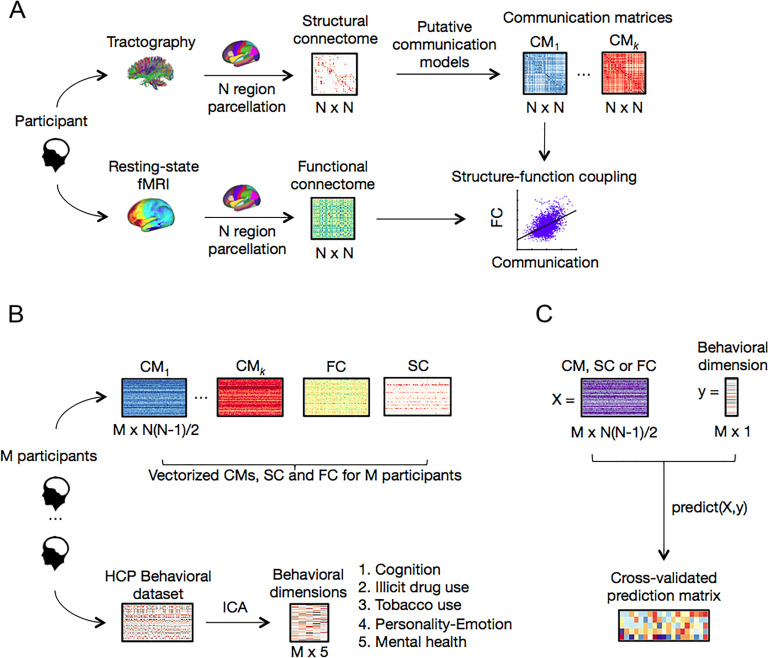
Methodology overview. (A) For each participant in our sample, structural connectomes comprising *N* cortical regions were mapped using white matter tractography applied to diffusion MRI. Structural connectivity matrices were transformed into communication matrices *C* ∈ℝ^*N*×*N*^, where *C*(*i*,*j*) denotes the communication efficiency from region *i* to region *j*. For each participant, a total of 15 communication matrices were derived representing different combinations of network communication models (shortest paths, navigation, diffusion, search information, communicability) and connection weight definitions (binary, weighted, distance). To assess structure-function coupling, communication matrices were correlated with FC matrices computed from resting-state functional MRI data. (B) Communication, FC, and SC matrices were vectorized and aggregated across *M* = 889 participants, resulting in 17 *M* × *N*(*N* − 1)/2 matrices of explanatory variables. A set of five behavioral dimensions was computed by applying independent component analysis (ICA) to the HCP dataset of behavioral phenotypes. (C) Communication, SC, and FC matrices were used to predict behavior. An entry in the resulting 5 × 17 prediction matrix corresponds to the mean cross-validated association between a communication or connectivity matrix and a behavioral dimension.

Connectome mapping yielded a structural connectivity (SC) matrix for each individual. These matrices represented connectivity between directly connected regions and were generally sparse because of an absence of white matter tracts between a majority of region pairs. To model the impact of polysynaptic neural signaling, each individual’s connectivity matrix was transformed into a communication matrix (Figure 1A). Communication matrices were of the same dimension as the SC matrices, but fully connected in most cases, and they quantified the efficiency of communication between indirectly (polysynaptic) as well as directly connected pairs of regions under a given network communication model. In contrast, the SC matrices only characterized directly connected pairs of regions.

We considered three connectivity weight definitions: (a) *weighted:* connection weights defined as the number of tractography streamline counts between regions; (b) *binary:* non-zero connection weights set to 1; and (c) *distance:* nonzero connection weights set to the Euclidean distance between regions. Network communication models computed on these connectomes operationalize metabolic factors conjectured to shape large-scale signaling: (a) adoption of high-volume white matter projections that putatively enable fast and reliable signal propagation (weighted); (b) reduction of the number of synaptic crossings (binary); and (c) reduction of the physical length traversed by signals (distance; Bullmore & Sporns, 2012; Fornito et al., 2016; Rubinov & Sporns, 2010).

### Predicting Behavior With Models of Connectome Communication

Statistical models were trained to independently predict five dimensions of behavior (cognition, illicit substance use, tobacco use, personality-emotional traits, mental health) based on features comprising an individual’s communication matrix (Figures 1B, 1C). Training and prediction were performed separately for a total of 15 communication matrices representing different connection weight definitions (binary, weighted, distance) and network communication models (shortest paths, navigation, diffusion, search information, communicability). Additionally, predictions based on an individual’s SC and FC were computed to provide accuracy benchmarks. The five behavioral components represent orthogonal dimensions that were parsed from a comprehensive set of behavioral measures using independent component analysis (see the Methods section).

**Figure 2.**
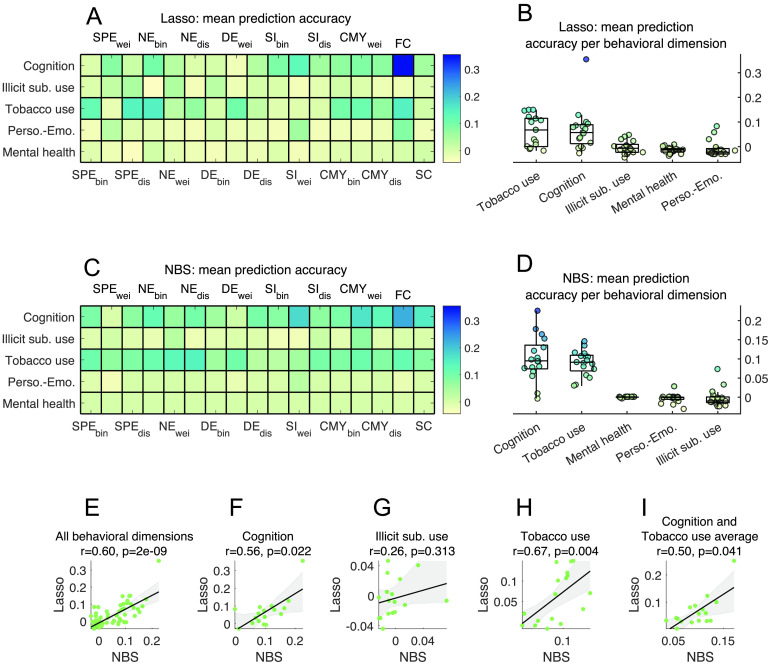
Predicting individual variation in human behavioral dimensions using models of connectome communication, (*N* = 360 thresholded connectomes). (A) Matrix of Pearson correlation coefficients between observed and predicted behavior. Lasso regression was used to predict five orthogonal behavioral dimensions (rows) from 15 connectome communication models, as well as SC and FC (columns, predictors). (B) Lasso regression prediction accuracies stratified by behavioral dimensions. Each boxplot summarizes a row of the prediction accuracy matrix and the superimposed data points are colored accordingly. Top and bottom edge boxplots indicate, respectively, the 25th and 75th percentiles, while the central mark shows the distribution median. Mean prediction accuracies significantly differed between the five behavioral dimensions (*F*_(4,80)_ = 10.67, *p* = 5 × 10^−7^). (C–D) Same as (A–B), but for predictions carried out using a regression model based on features identified by the NBS. Again, mean prediction accuracies were significantly different between behavioral dimensions (*F*_(4,80)_ = 47.18, *p* = 2 × 10^−20^). Scatterplots showing the association (Spearman rank correlation coefficient and *p* value) between lasso and NBS prediction accuracies for (E) all behavioral dimensions, (F) cognition, (G) illicit substance use, (H) tobacco use, and (I) the average between cognition and tobacco use prediction accuracies. SPE: shortest path efficiency, NE: navigation efficiency, DE: diffusion efficiency, SI: search information, CMY: communicability, bin: binary, wei: weighted, dis: distance.

Out-of-sample prediction accuracy was evaluated for 10 repetitions of a tenfold cross-validation scheme. The Pearson correlation coefficient between the actual and out-of-sample predicted behavior was used to quantify prediction accuracy for each behavioral dimension. To ensure that our results were not contingent on the adoption of a particular statistical model, predictions were independently performed using lasso regression (Tibshirani, 1996) and a regression model based on features identified by the network-based statistic (NBS; Zalesky, Fornito, & Bullmore, 2010; see the Methods section). Prediction accuracies were averaged across cross-validation folds and repetitions, and visualized in the form of a matrix comprising behavioral dimensions (rows) and communication models (columns; Figures 2A, 2C).

We found that individual variation in some behavioral dimensions could be predicted with greater accuracy than others (lasso: *F*_(4,80)_ = 10.67, *p* = 5 × 10^−7^; NBS: *F*_(4,80)_ = 47.18, *p* = 2 × 10^−20^). Dimensions characterizing cognition (respective lasso and NBS accuracies averaged across all predictors: 0.068, 0.101) and tobacco use (0.061, 0.089) could be predicted more accurately on average, whereas comparably weaker predictions of illicit substance use (−0.003, −0.002), personality-emotion (−0.008, −0.003), and mental health (−0.014, −0.0003) were evident (Figures 2B, 2D).

Prediction accuracies were consistent between the two statistical models (NBS, lasso), both when pooling the five behavioral dimensions (Spearman rank correlation coefficient *r*_(83)_ = 0.60, *p* = 2 × 10^−9^; Figure 2E), as well as separately for cognition (*r*_(16)_ = 0.56, *p* = 0.022; Figure 2F) and tobacco use (*r*_(16)_ = 0.67, *p* = 0.004; Figure 2H). Lasso and NBS diverged for the dimensions that were less accurately predicted (e.g., *p* = 0.313 for illicit substance use; Figure 2G).

Focusing on lasso regression, we sought to determine whether behavioral predictions were robust to variations in our methodological settings. First, we found that adopting the mean square error to quantify predictive utility led to accuracies significantly associated with the ones computed based on Pearson correlation (Supporting Information, Figure S1). Second, we tested whether prediction accuracies were sensitive to changes in our connectome mapping pipeline. To this end, we recomputed behavioral predictions for three additional sets of connectomes: (a) *N* = 360 regions without connection thresholding, (b) *N* = 68 regions with connection thresholding, and (c) *N* = 68 regions without connection thresholding (see the Methods section). Prediction accuracies were typically significantly correlated across low- and high-resolution, as well as thresholded and unthresholded, connectomes (Supporting Information, Figure S2). More specifically, consistency across connectome mapping pipelines was strong when considering predictions pooled across all five behavioral dimensions and relatively modest when focusing on cognition and tobacco use, indicating a potential effect of parcellation and connection thresholding to the predictive utility of different communication models.

Together, these findings suggest that network communication models (as well as SC and FC) can explain out-of-sample interindividual variance in behavior. More specifically, cognition and tobacco use were the most accurately predicted behavioral dimensions. For this reason, we henceforth focus subsequent analyses on the averaged prediction accuracy obtained for the cognition and tobacco use dimensions. This provides us with a single measure of how connectome communication relates to behavior by considering only the behavioral traits that can be predicted with relevant accuracy. The obtained prediction accuracy average was also consistent across the lasso and NBS methods (*r*_(16)_ = 0.50, *p* = 0.041; Figure 2I).

### Communication Models Improve the Behavioral Predictive Utility of the Human Connectome

**Figure 3.**
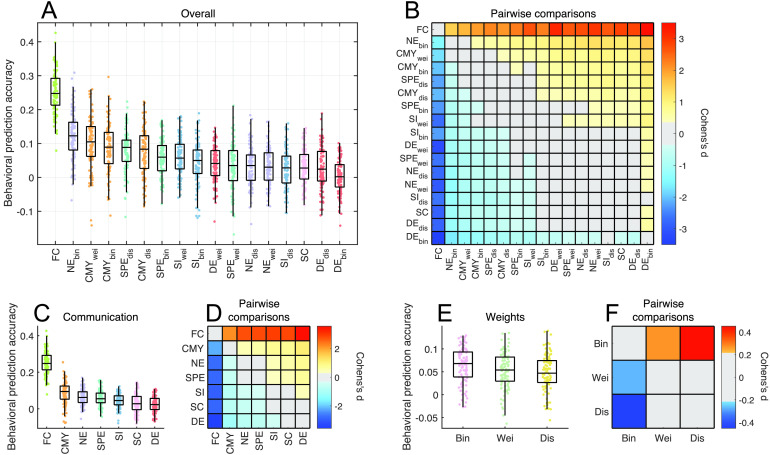
Comparison of the behavioral predictive utility of connectome communication models (Lasso regression, *N* = 360 thresholded connectomes, average cognition and tobacco use prediction accuracies). Across panels, top and bottom edge boxplots indicate, respectively, the 25th and 75th percentiles, while the central mark shows the distribution median. (A) Prediction accuracy distributions for 10 repetitions of tenfold cross validation. Communication models, SC, and FC were sorted based on their median prediction accuracy. (B) Effect size matrix of pairwise statistical comparisons between predictors. Warm- and cool-colored cells indicate predictor pairs with significantly different means, as assessed by a repeated-measures *t* test (Bonferroni-corrected for 136 multiple comparisons with significance threshold *α* = 3.67 × 10^−4^). A warm-colored *i*,*j* matrix entry indicates that predictor *i* yields significantly more accurate predictions than predictor *j*. (C) Prediction accuracy distributions of communication models averaged across connection weight definitions. SC and FC were not subjected to averaging and accuracies remain the same as in panel (A). (D) Effect size matrix of pairwise repeated-measures *t* tests between distributions in panel (C), with colored cells indicating significant differences in mean prediction accuracies (Bonferroni-corrected with significance threshold *α* = 0.0024). (E) Prediction accuracy distributions of connectomes with different connection weight definitions averaged across communication models. (F) Same as panel (D), but Bonferroni-corrected with significance threshold *α* = 0.0167.

We sought to compare communication models, as well as SC and FC, in terms of their behavioral prediction accuracy. Figure 3A shows the distributions of out-of-sample accuracies (10 repetitions of tenfold cross validation, averaged for the cognition and tobacco use dimensions) obtained for the each predictor using lasso regression. Accuracy distributions were ranked based on their medians. FC (median accuracy: 0.24) provided markedly greater accuracy than all communication models and SC. Binary navigation (median accuracy: 0.12) and weighted communicability (median accuracy: 0.10) followed as the second and third most predictive communication models. Crucially, we observed that the majority of communication models yielded greater prediction accuracy than SC (median accuracy: 0.03). This indicates that modeling polysynaptic signaling through the transformation of SC into communication matrices improved the behavioral predictive utility of structural connectomes.

We performed repeated measures *t* tests to assess pairwise statistical differences in the predictive utility of communication models and connectivity measures. Figure 3B shows the effect size matrix (Cohen’s *d*; Bonferroni-corrected for 136 multiple comparisons with significance threshold *α* = 3.67 × 10^−4^) of differences between mean prediction accuracies, with warm- and cool-colored cells indicating model pairs for which a significant difference was observed. As expected, FC outperformed all other predictors (e.g., *p* = 1 × 10^−26^ between FC and binary navigation). The lack of colored cells along the main diagonal of the effective size matrix indicates that predictors of similar ranking seldom yielded significantly different accuracy. Importantly, seven communication models (out of 15) significantly outperformed SC, including binary navigation; binary, weighted, and distance communicability; binary and distance shortest paths; and weighted search information (all *p* < 10^−4^). This underscores the improvement in behavioral predictive utility gained from accounting for polysynaptic communication in structural connectomes, compared with predictions that only account for direct structural connections. The magnitude of statistical differences between communication models was better visualized when plotting effect size matrices excluding comparisons to FC (SupportingInformation, Figure S3).

Importantly, the behavioral prediction accuracies reported in Figure 3A were significantly larger than those obtained by computing network communication models on null sets of topologically randomized connectomes (Supplementary Note 1; Supporting Information, FigureS4). This corroborates the notion that network communication models are capable of predicting interindividual variation in human behavior, and that observed differences in prediction accuracies reflect meaningful distinctions in the predictive utility of different models. Additionally, we found that the pairwise comparisons between models shown in Figure 3B were stable across each of the 10 repetitions of the performed tenfold cross validation (Supplementary Note 2; Supporting Information, Table S1).

Next, we aimed to separate the effects of communication model choice and connection weight definition on prediction accuracy. To this end, accuracies were averaged over the three weight definitions for each communication model (Figures 3C, 3D), or averaged over the 15 models for each weight definition (Figures 3E, 3F). Prediction accuracies for FC and SC, which were not computed for multiple weight definitions, remained the same as shown in Figure 3A. With respect to the effect of communication model, we found that communicability significantly outperformed other models and SC (e.g., *p* = 3 × 10^−5^,2 × 10^−11^ for comparisons of communicability to navigation and SC, respectively), although FC remained the leading predictor. Navigation and shortest paths featured in second and third positions, both performing better than SC (*p* = 3 × 10^−7^,3 × 10^−5^, respectively) and with no statistical difference between them (*p* = 0.26). With respect to connection weight definition, binary connectomes yielded significantly higher prediction accuracies, on average, compared with weighted and distance connectomes (*p* = 0.009,2 × 10^−5^, respectively), albeit with a weaker effect size than differences between communication models. This suggests that the choice of communication model may be more important to behavior predictions than the definition of connection weights.

To gain further insight into these results, we executed additional analyses in which we considered predictions for the cognition and tobacco use dimensions separately (Supplementary Note 3; Supporting Information, Figures S5, S6). While these investigations reiterated the overall good performance of navigation and communicability, they also revealed the presence of certain dimension-specific relationships between communication and behavior. For instance, search information yielded top- and bottom-ranking predictions for cognition and tobacco use, respectively.

Finally, aiming to assess the robustness of our findings to the choice of prediction method, we analyzed behavioral predictions derived using a regression model applied to features identified by the NBS. As with the lasso, we examined NBS predictions combined across the cognition and tobacco use (Supporting Information, Figure S7), as well as for each of these dimension separately (Supporting Information, Figures S8, S9). As previously reported in Figure 2, NBS and lasso prediction accuracies were significantly correlated. FC remained the strongest predictor of behavior, although with a smaller margin of difference to navigation and communicability. Despite this overall agreement, we observed that SC yielded higher ranking predictions under the NBS method (5th highest ranking predictor) than the lasso (15th predictor). Interestingly, SC’s performance under the NBS diverged widely between the cognition (4th predictor) and tobacco use components (16th predictor), reiterating the dimension-specific character of brain-behavior relations observed in the lasso results. It is worth noting that the NBS feature selection process is better suited to sparse graphs (Zalesky et al., 2010), which could confer a predictive advantage to sparse SC matrices over fully connected communication and FC matrices.

Taken together, the behavioral prediction analyses led to three key findings. First, behavioral predictions were more accurate when performed based on functional rather than structural attributes. Second, while navigation and communicability typically showed high predictive utility, our results did not point towards a single communication model as the best predictor of human behavior. This indicates that different communication models may be better suited to predict different behavioral dimensions, possibly suggesting the presence of behavior-specific signaling mechanisms in the human brain. Third, the transformation of SC (only direct connections) into communication matrices (models of polysynaptic interactions) typically led to an improvement of structural-based predictions, bringing them closer to the predictive utility of FC. Importantly, the magnitude of this improvement, as well as which and how many communication models conferred predictive benefits, varied depending on behavioral dimensions and prediction methods. Collectively, these findings indicate that connectome communication models capture higher order structural relations among brain regions that can better account for interindividual variation in behavior than SC alone.

### Communication Models Improve Structure-Function Coupling

We next investigated whether accounting for network communication in the structural connectome can improve the strength of the relation between SC and FC, known as structure-function coupling. Classically, associations have been directly tested between structural and functional connections (Honey, Kötter, Breakspear, & Sporns, 2007). A growing body of work indicates that accounting for higher order regional interactions through models of polysynaptic signaling (i.e., transforming structural connectomes into communication matrices) can improve structure-function coupling (Abdelnour, Voss, & Raj, 2014; Goñi et al., 2014; Mišić et al., 2015; Seguin et al., 2019; Suárez et al., 2020). For two regions that are not directly connected with an anatomical fiber, strong FC is conjectured to indicate the presence of an efficient signaling path that facilitates communication through the underlying anatomical connections (Avena-Koenigsberger et al., 2018).

To test this hypothesis, we computed the association between FC and communication matrices for each individual in our sample. Additionally, as benchmarks, we also considered the association of FC to SC and to interregional Euclidean distance. Associations were computed as the Spearman correlation between upper triangular matrix entries. In addition to individual-level associations, we also analyzed structure-function coupling derived from group-level SC and FC. Finally, associations were derived for coarse- (*N* = 68 regions) and fine-grained (*N* = 360 regions) connectomes, which were thresholded prior to the computation of communication models. FC matrices were not thresholded. Further details on the computation of structure-function coupling are provided in the Methods section.

**Figure 4.**
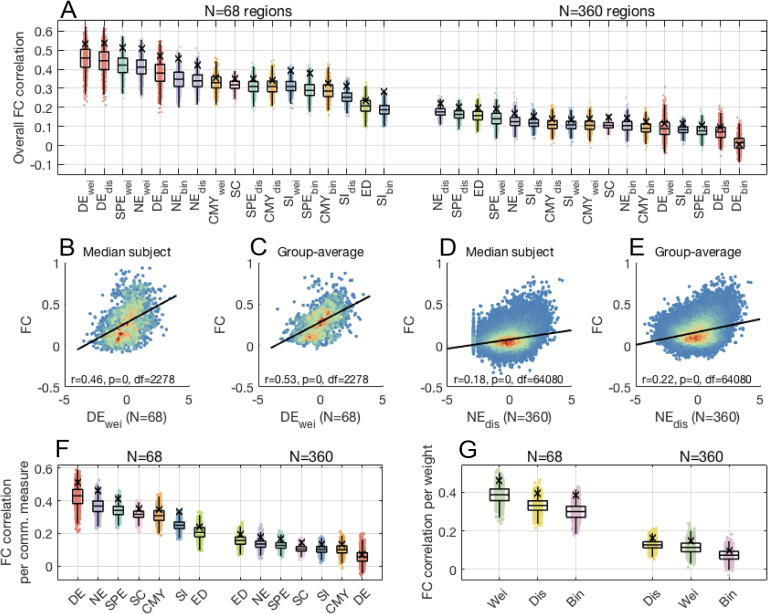
Structure-function coupling across connectome communication models (*N* = 68,360 thresholded connectomes). (A) Data points show individual-level correlation of FC to communication, SC, and Euclidean distance matrices. Black crosses indicate correlations obtained for group-averaged matrices. Top and bottom edge boxplots indicate, respectively, the 25th and 75th percentiles, while the central mark shows the distribution median. Communication models, SC, and Euclidean distance were ranked according to the median structure-function coupling strength across individuals. (B) Scatterplot depicting the relationship between FC and the top-ranked communication model for connectomes comprising *N* = 68 regions, for the median individual. For ease of visualization, communication matrix entries were resampled to normal distributions. Warm and cool colors indicate high and low data point density, respectively. (C) Same as (B), but for group-average matrices. (D–E) Same as (B–C), but for connectomes comprising *N* = 360 regions. (F) Structure-function coupling for communication models, SC, and Euclidean distance, averaged across connection weight definitions. (G) Structure-function coupling obtained for binary, weighted, and distance connectomes, averaged across communication models.

As previously reported (Goñi et al., 2014), communication matrices were correlated with FC, irrespective of the particular communication model (Figure 4). In other words, FC was generally stronger between regional pairs interconnected by more efficient communication pathways. Group-level correlations (*r*_*G*_; black crosses) were universally stronger than those obtained for the median individual (*r*_*I*_; boxplots), supporting the notion that predicting population-level FC traits is less challenging than modeling idiosyncratic relationships between brain structure and function.

We found that parcellation resolution had a strong influence on the strength of structure-function coupling. The link between structure and function weakened for high-resolution connectomes, irrespective of the communication model (Figure 4A). Moreover, the ranking of communication models in terms of structure-function coupling differed between connectome resolutions (Spearman rank correlation between low- and high-resolution FC predictions *p* = 0.65). For *N* = 68 regions, weighted and distance diffusion yielded the strongest structure-function couplings (*r*_*I*_ = 0.46 and *r*_*G*_ = 0.53 for weighted diffusion; Figures 4B, 4C). This recapitulates previous work indicating the functional predictive utility of random walk models applied to connectomes comprising less than 100 regions (Abdelnour et al., 2014). However, in sharp contrast, diffusion performed poorly for *N* = 360 regions, going from yielding the most accurate estimates of FC in low resolution to ranking as the worst overall predictor in high resolution. Conversely, the coupling between Euclidean distance and FC showed the opposite relationship to connectome resolution, with interregional distances leading to weak and strong associations for coarse- and fine-grained parcellations, respectively.

Navigation and shortest paths resulted in consistently high-ranked FC predictions regardless of connectome resolution. For *N* = 68 regions, weighted navigation and shortest paths showed comparable associations with the top-ranking diffusion models (e.g., *r*_*I*_ = 0.42 for weighted shortest paths). For *N* = 360 regions, distance navigation was the top-ranking model (*r*_*I*_ = 0.18 and *r*_*G*_ = 0.22; Figures 4D, 4E), followed by distance shortest paths in second place, both outperforming the Euclidean distance benchmark in the third position.

Crucially, despite the effects of connectome resolution, modeling polysynaptic communication on top of structural connectomes tightened structure-function coupling. This was the case for 8 and 9 out of the 15 communication models considered, for low- and high-resolution connectomes, respectively. For instance, for the median individual, weighted diffusion in 68-region connectomes strengthened coupling by 46% compared with SC, while computing distance navigation in 360-region connectomes boosted FC predictions by 66% compared with SC.

Grouping functional predictions by communication models reiterated differences between low- and high-resolution connectomes (Figure 4F). Grouping predictions by connection weight definitions showed that, on average, communication models computed on weighted and distance connectomes led to stronger associations for coarse- and fine-grained parcellations, respectively (Figure 4G), suggesting that the established influence of interregional distance in SC and FC (Alexander-Bloch et al., 2013; Roberts et al., 2016) may be stronger for connectomes derived at finer levels of areal granularity.

In summary, we observed that structure-function coupling is affected by connectome resolution and by whether associations are computed on individual or population levels. Regardless of parcellation granularity, most connectome communication models contributed to strengthening structure-function coupling. Moreover, navigation and shortest paths yielded the most accurate and reliable predictions of FC. While here we focused on thresholded connectomes, similar results were observed for unthresholded networks (Supporting Information, Figure S10). Rankings of functional predictive utility also remained consistent when stratifying analyses between structurally connected and unconnected region pairs, as well as for intrahemispheric structure-function associations (Supplementary Note 4; Supporting Information, Figure S11). Together, these observations build on the behavioral prediction findings, further supporting the notion that connectome communication models contribute to bridging the gap between brain structure and function.

### Ranking Communication Models

**Figure 5.**
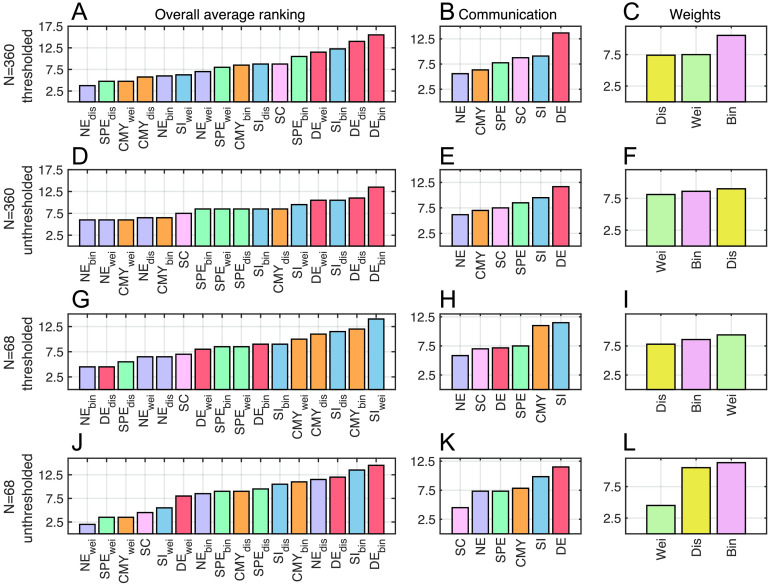
Rankings of communication models combining behavioral and functional predictions. Overall, communication, and weight definition rankings for (A–C) *N* = 360 thresholded, (D–F) *N* = 360 unthresholded, (G–I) *N* = 68 thresholded, and (J–L) *N* = 68 unthresholded connectomes.

Finally, we derived a combined ranking of predictive utility, as the average of behavioral and functional prediction rankings, for the 15 communication models explored and SC (Figure 5). This was performed for the four connectome mapping pipelines explored in our analyses. Behavioral and functional results were given equal weight in the combined rankings. For *N* = 360 thresholded connectomes, the only case in which behavioral analyses were carried out using both lasso and NBS prediction methods, a weighted average assigning 0.25 weight to lasso behavioral rankings, 0.25 weight to NBS behavioral rankings, and 0.5 weight to structure-function coupling rankings was applied.

For *N* = 360 thresholded connectomes, we found that distance navigation showed the highest combined predictive utility (average ranking *τ* = 3.7; Figure 5A), followed by a tie between distance shortest paths and weighted communicability (*τ* = 4.7). SC featured in the 11th position (*τ* = 8.7) and was outranked by most navigation, communicability, shortest paths, and search information models.

Contrasting combined rankings across connectome mapping pipelines led to several interesting observations. First, *N* = 360 thresholded and unthresholded connectomes led to the only pair of significantly correlated overall rankings (Spearman rank correlation *r*_(15)_ = 0.65, *p* = 0.007). This corroborates our previous findings that parcellation choice and connection density influence the predictive utility of network communication models. Second, network communication models were more beneficial when computed on high-resolution and thresholded connectomes. Intuitively, a densely connected network requires few polysynaptic signaling paths, since most regions can communicate via direct connections. This renders the transformation of SC into communication matrices less relevant, and therefore less advantageous for predictions. Third, combined rankings did not provide a clear picture on what connection weight definition may be more relevant for behavioral and functional predictions (Figures 5C, 5F, 5I, 5L). Fourth, for all connectome mapping pipelines, we found certain communication models that improved the predictive utility of the human connectome. In the case of unthresholded *N* = 68 connectomes, the scenario in which communication models provide the least benefits, we still observed that weighted navigation, shortest paths, and communicability outranked SC. Fifth, navigation was the top-ranking model across all connectome mapping pipelines. This was the case when considering overall rankings (Figures 5A, 5D, 5G, 5J) as well as rankings grouped by communication models (Figures 5B, 5E, 5H, 5K). Weighted navigation outranked SC in all explored scenarios, and excluding unthresholded *N* = 68 connectomes, navigation outranked SC regardless of connection weight definitions.

Collectively, these results indicate that despite differences across connectome mapping pipelines, certain network communication models improved the combined behavioral and functional predictive utility of the human connectome. In particular, navigation was consistently positioned as the highest ranking model, indicating that the transforming of SC into navigation communication matrices is reliably advantageous for predictions of human behavior and FC.

## DISCUSSION

Human cognition and behavior arise from the orchestrated activity of multiple brain regions (Friston, 2002; Laughlin & Sejnowski, 2003). Resting-state FC is currently one of the most widely used neuroimaging measures to quantify this concerted activity (Cole, Ito, Bassett, & Schultz, 2016; Sripada et al., 2019; Yeo et al., 2011). It is thus unsurprising that statistical methods trained on functional brain networks led to the most accurate predictions of human behavior. Importantly, the signaling processes that facilitate synchronous interregional activity must unfold along structural connections forming direct or indirect (polysynaptic) communication paths. Therefore, brain structure, brain function, neural communication, and human behavior are tightly intertwined. This is corroborated by the key conclusion of the present study: Accounting for polysynaptic communication in SC matrices can substantially improve structure-function coupling and the predictive utility of SC. While accounting for communication did not lead to SC outperforming FC with respect to behavior prediction, it narrowed the gap between the predictive utility of the two connectivity modalities.

As investigators tackle the long-standing challenge of elucidating the relationship between brain structure and function (Amico & Goñi, 2018; Damoiseaux & Greicius, 2009; Park & Friston, 2013), it has become increasingly clear that FC arises from high-order regional interactions that cannot be explained by direct anatomical connections (Suárez et al., 2020). In line with this notion, we found that taking polysynaptic signaling into account through network communication models strengthened structure-function coupling. This observation recapitulates earlier reports on the functional predictive utility of connectome communication models (Goñi et al., 2014) and provides support to the notion that FC is facilitated by communication pathways in the underlying structural connectome. Taken together, the behavioral and functional prediction analyses contribute empirical evidence that connectome communication models act as a bridge between structural and functional conceptualizations of brain networks (Avena-Koenigsberger et al., 2018; Mišić, Goñi, Betzel, Sporns, & McIntosh, 2014).

Importantly, brain structure-function relationships encompass a rich and diverse field of research, with several alternative classes of higher order models showing promise in modeling function from structure. Examples include biophysical models of neural activity (Breakspear, 2017; Deco, Kringelbach, Jirsa, & Ritter, 2017; Sokolov et al., 2018), statistical methods (Messé, Rudrauf, Benali, & Marrelec, 2014; Mišić et al., 2016), and other approaches centered around network communication that we did not explore in the present work (Kuceyeski, Jamison, Owen, Raj, & Mukherjee, 2019; Mišić et al., 2015; Osmanloğlu et al., 2019; Raj, Kuceyeski, & Weiner, 2012; Vázquez-Rodríguez, Liu, Hagmann, & Mišić, 2020). Likewise, relating neuroimaging data to behavior is a central goal of neuroscience (Medaglia, Lynall, & Bassett, 2015; Mišić & Sporns, 2016). Recent studies have explored neural correlates of behavior and cognition by leveraging graph measures of brain organization (Bertolero, Yeo, Bassett, & D’Esposito, 2018; van den Heuvel, Stam, Kahn, & Hulshoff Pol, 2009), dynamic patterns of FC fluctuations (Liégeois et al., 2019; Pedersen, Zalesky, Omidvarnia, & Jackson, 2018), multivariate correlation methods (Perry et al., 2017; Smith et al., 2015), and machine learning techniques (He et al., 2020; Li et al., 2019). Our analyses sought to complement these efforts from the perspective of connectome communication.

We reiterate that the goal of this paper was not to show that network communication models lead to more accurate predictions than alternative approaches, nor that our prediction scheme and statistical methods are superior to previously adopted techniques. Rather, we were interested in comparing the predictive utility of candidate models of connectome communication, as well as connectivity and distance benchmarks, in a controlled and internally consistent manner. Similarly, although we explored multiple brain network reconstruction pipelines, we were not primarily concerned with which mapping techniques produced connectomes with the highest predictive utility. The choice of parcellation schemes (Eickhoff, Yeo, & Genon, 2018) and whether to threshold structural connectomes (Buchanan et al., 2020; Civier, Smith, Yeh, Connelly, & Calamante, 2019) are both challenging open questions that fall outside the scope of this work.

### Comparisons Between Connectome Communication Models

Communication matrices computed with the navigation and communicability models typically led to the highest ranking behavioral predictions among the candidate signaling strategies explored. It is important to notice, however, that search information, shortest paths, and SC also performed well in certain scenarios. Therefore, while our behavioral results suggested the benefits of modeling polysynaptic signaling, they did not provide a clear answer to the question of which communication models are most associated with human behavioral dimensions. Alternatively, our findings may indicate the interesting possibility that large-scale information integration in the brain is not facilitated by a unique signaling mechanism, and that different communication models may find more utility in describing varied behavioral and cognitive processes.

Navigation and shortest paths led to the most reliable FC predictions, featuring as the best models for high-resolution connectomes and closely following behind diffusion for low-resolution connectomes. Navigation and shortest paths computed on distance connectomes led to FC predictions that surpassed those obtained from Euclidean distance, which exerts a well-documented influence on both SC and FC (Alexander-Bloch et al., 2013; Roberts et al., 2016; Vértes et al., 2012). Furthermore, given the high efficiency of communication along navigation and shortest paths, these findings suggest that FC is facilitated primarily by efficient signaling pathways. This observation stands in contrast with previous work on the strong functional relevance of models that incorporate deviations from optimal routes, such as search information (Betzel et al., 2019; Goñi et al., 2014) and communicability (Osmanloğlu et al., 2019), underscoring the importance of further research on the validation of network communication models.

We evaluated network communication models across a wide range of methodological scenarios, including different gray matter parcellations, connection density thresholds, statistical learning methods, and behavioral dimensions. While our results were stable for certain combinations of these factors, we found that the predictive utility of communication models substantially varied between some scenarios. This variability makes it difficult to draw strong conclusions about the extent to which different models are useful to understanding biological neural signaling patterns. Nonetheless, combining behavioral and functional prediction rankings consistently positioned navigation as the top-ranking model across connectome mapping pipelines. These findings contribute to the growing body of work supporting the neuroscientific utility of network navigation (Allard & Serrano, 2020; Pappas, Craig, Menon, & Stamatakis, 2020; Seguin et al., 2018; Wang et al., 2019) and highlight the potential of this signaling strategy as a model of information transfer in nervous systems.

In addition to investigating putative neural signaling strategies, we also considered different connection weight definitions. Polysynaptic transmission of neural signals entails metabolic expenditures related to the propagation of action potential along axonal projections and the crossing of synaptic junctions. Communication in the brain is thought to be metabolically frugal (Bullmore & Sporns, 2012; Laughlin & Sejnowski, 2003), but what aspects of structural connectivity are relevant to energy consumption in large-scale signaling remain unclear. We found that weighted and distance connectomes typically led to communication matrices with higher predictive utility. This is initial evidence that neural signaling may favor communication paths prioritizing the adoption of physically short and high-volume connections, instead of paths that reduce the number of synaptic crossings between regions. Additionally, these observations warrant further investigation of the relatively unexplored distance connectome (Stiso & Bassett, 2018).

Previous evidence that connection thresholding is an unnecessary step in brain network analyses is primarily based on studies considering weighted connectomes (Civier et al., 2019), for which interregional connectivity strength is determined as a function of streamline counts or fractional anisotropy (Sotiropoulos & Zalesky, 2019). Along these lines, we found that connectomes weighted by streamline counts led to the best performing predictors when considering unthresholded brain networks. However, we note that two thirds of the communication models explored in our analyses were based on binary and distance connectomes, which do not contain information on streamline counts. Therefore, it is expected that connection thresholding would impact the overall rankings of communication models.

In accordance with previous reports (Messé, 2020; Messé, Rudrauf, Giron, & Marrelec, 2015), we observed that FC predictions were more accurate for low- rather than high-resolution connectomes, as well as for group- rather than individual-level analyses. This is not surprising since the number of functional connections grows quadratically with the number of regions and capturing idiosyncrasies in FC is more challenging than modeling general principles of connectivity. Despite their simplicity, these observations are important to the validation of FC prediction methods, suggesting that models constructed and evaluated on coarse and population-level networks may not generalize to more challenging settings.

### Limitations and Future Directions

Several methodological limitations of the present work should be discussed. First, given the sensitivity of model rankings to some aspects of connectome mapping pipelines, further work exploring alternative brain network reconstruction methods is necessary. For instance, validation of our results for connectomes mapped using probabilistic tractography and/or larger numbers of streamline seeds would be valuable. We also note that white matter tractography algorithms are susceptible to a number of known biases that could potentially impact our findings (Maier-Hein et al., 2017).

A conceptual limitation of our behavioral analyses was that model selection was performed on the same data used to evaluate the accuracy of single models (out-of-sample test set). In addition, we note that behavioral prediction accuracy estimates from different cross-validation folds are not independent from each other. This is a limitation of the statistical tests performed to compare the utility of communication models. Once again, these observations underscore the need for additional efforts to evaluate network communication models using alternative datasets and machine learning methods.

Another interesting future research direction is to investigate the contributions of specific brain regions to the predictive utility of different communication models. This could be achieved by examining lasso regression weights and NBS connected components. Alternatively, behavior and functional predictions could be performed based on region-wise communication efficiencies, rather than complete communication matrices (Vázquez-Rodríguez et al., 2019). Efforts in these directions could help elucidate how different communication models utilize features of connectome topology to facilitate information transfer.

While we sought to evaluate a wide range of communication models, alternative network propagation strategies could provide valuable insight into mechanisms of neural signaling and warrant further research. These include linear transmission models (Mišić et al., 2015), biased random walks (Avena-Koenigsberger et al., 2019), cooperative learning (Tipnis, Amico, Ventresca, & Goñi, 2018), dynamic communication models (Gilson et al., 2019), and information-theoretic approaches (Amico et al., 2019).

In conclusion, we demonstrated that taking into account polysynaptic signaling via models of network communication can improve the behavioral and functional predictive utility of the human structural connectome. This work contributes to our understanding of which network communication strategies may be more useful as large-scale neural signaling models, providing novel insights to researchers interested in characterizing information processing in nervous systems.

## METHODS

### Structural Connectivity Data

Minimally preprocessed high-resolution diffusion-weighted magnetic resonance imaging (MRI) data were obtained from the Human Connectome Project (HCP; Van Essen et al., 2013). Details about the acquisition and preprocessing of diffusion MRI data are found in Glasser et al. (2013); Sotiropoulos et al. (2013). Analyses were restricted to participants with complete HCP 3T imaging protocol, yielding a total sample of 889 healthy adults (age 22–35, 52.8% females). Whole-brain structural connectomes were mapped using diffusion tensor imaging and a deterministic white matter tractography pipeline implemented using MRtrix3 (Tournier, Calamante, & Connelly, 2012; FACT tracking algorithm, 5 × 10^6^ streamlines, 0.5-mm tracking step-size, 400-mm maximum streamline length, and 0.1 fractional anisotropy cutoff for termination of tracks). Deterministic tractography is less prone to false positive than alternative reconstruction approaches (Maier-Hein et al., 2017; Sarwar, Ramamohanarao, & Zalesky, 2019), which leads to connectomes that may be better suited for network and graph-theoretical analyses (Zalesky et al., 2016). The connection weight between a pair of regions was defined as the total number of streamlines connecting them, resulting in an *N* × *N* weighted connectivity matrix for each participant. Group-level structural connectomes were computed by averaging the connectivity matrices of all subjects.

We used cortical parcellations containing *N* = 68,360 regions. The 68-region parcellation consists of the anatomically delineated cortical areas of the Desikan-Killiany atlas (Desikan et al., 2006). The 360-region parcellation is a multimodal atlas constructed from high-resolution structural and functional data from the HCP (Glasser et al., 2016). We also considered thresholded and unthresholded connectomes. Following connection density thresholding, only the top 15% and 20% strongest connections (in terms of streamline counts) were kept in connectomes comprising 360 and 68 regions, respectively. Connection density thresholds were chosen as the (approximate) lowest values that resulted in nonfragmented brain networks for all subjects in our sample. Unthresholded connectomes maintained all connections identified in the structural connectivity reconstruction process.

### Connection Weight and Length Definitions

A structural connectome can be defined in terms of a *N* × *N* adjacency matrix of connectivity weights (*W*) or lengths (*L*). Connection weights provide a measure of the strength and reliability of anatomical connections between region pairs, while connection lengths quantify the distance or travel cost between region pairs. Different network communication measures are computed based on *W* (e.g., diffusion efficiency and communicability), *L* (e.g., shortest path efficiency and navigation efficiency), or a combination of both (e.g., search information).

We considered three definitions of *W*: weighted, binary, and distance. In the weighted case, *W*_*wei*_(*i*,*j*) was defined as the total number of streamlines with one endpoint in region *i* and the other in region *j*. The binary adjacency matrix was defined as *W*_*bin*_(*i*,*j*) = 1 if *W*_*wei*_(*i*,*j*) > 0 and *W*_*bin*_(*i*,*j*) = 0 otherwise. Distance-based connectivity was defined as *W*_*dis*_(*i*,*j*) = 1/*D*(*i*,*j*) if *W*_*wei*_(*i*,*j*) > 0 and *W*_*dis*_(*i*,*j*) = 0 otherwise, where *D* is the Euclidean distance matrix between region centroids.

Similarly, *L* was also defined in terms of binary, weighted, and distance connection lengths. In all three cases, L(i,j)=∞ for *ij* region pairs that do not share a direct anatomical connection, ensuring that communication is restricted to unfold through the connectome. Binary (*L*_*bin*_) and distance-based (*L*_*dis*_) connection lengths are straightforwardly defined from their weighted counterparts as *L*_*bin*_(*i*,*j*) = 1 if *W*_*bin*_(*i*,*j*) = 1 and $L_{bin}(i,j)=\infty$ otherwise, and *L*_*dis*_(*i*,*j*) = *D*(*i*,*j*) if *W*_*dis*_(*i*,*j*) > 0 and $L_{dis}(i,j)=\infty$ otherwise. Lengths based on the streamline count between region pairs were computed by monotonic weight-to-length transformations that remap large connection weights into short connection lengths. This way, white matter tracts conjectured to have high caliber and integrity are considered to be faster channels of communication than weak and unreliable ones. We considered a logarithmic weight-to-length remapping such that *L*_*wei*_ = −*log*_10_(*W*_*wei*_/*max*(*W*_*wei*_) + 1) (the unity addition to the denominator avoids the remapping of the maximum weight into zero length; Seguin et al., 2018), producing normally distributed lengths that attenuate the importance of extreme weights (Avena-Koenigsberger et al., 2016; Rubinov, Ypma, Watson, & Bullmore, 2015).

### Network Communication Models

In this section, we provide details regarding the definition of the five network communication models evaluated in this study. All computations were carried out using freely available code provided in the Brain Connectivity Toolbox (https://sites.google.com/site/bctnet/; Rubinov & Sporns, 2010).

First, we note a subtle but important distinction between network communication models and measures. A network communication model (e.g., shortest path routing) provides a strategy or algorithm to transfer information between node pairs. In turn, a network communication measure (e.g., shortest path efficiency) quantifies, from a graph-theoretical standpoint, the efficiency of information transfer achieved by a given communication model. For simplicity, we used “model” throughout this paper to refer to both network communication models and measures.

We also note that certain communication measures are inherently asymmetric, in that *C*_*asy*_(*i*,*j*)≠*C*_*asy*_(*j*,*i*). While this asymmetry contains meaningful information on signaling properties of nervous systems (Seguin et al., 2019), in the present study we consider symmetric communication matrices given by *C*(*i*,*j*) = (*C*_*asy*_(*i*,*j*) + *C*_*asy*_(*j*,*i*))/2. This simplification allows us to take into account only the upper triangle of *C*, substantially reducing the dimensionality of our predictive models and contributing to the computational tractability of our analyses.

#### Shortest path efficiency.

Shortest path routing proposes that neural signaling takes place along optimally efficient paths that minimize the sum of connection lengths traversed between nodes. Let Λ^*^∈ℝ^*N*×*N*^ denote the matrix of shortest path lengths, where Λ^*^(*i*,*j*) = *L*(*i*,*u*) + … + *L*(*v*,*j*) and {*u*, …, *v*} is the sequence of nodes visited along the shortest path between nodes *i* and *j*. Shortest path efficiency was defined as *SPE* = 1/Λ^*^ (Latora & Marchiori, 2001). We computed binary (*SPE*_*bin*_), weighted (*SPE*_*wei*_), and distance (*SPE*_*dis*_) shortest path efficiency matrices based on *L*_*bin*_, *L*_*wei*_, *L*_*dis*_ connection length matrices, respectively.

#### Navigation efficiency.

Navigation routing identifies communication paths by greedily propagating information based on a measure of node (dis)similarity (Boguña et al., 2009). Following previous studies on brain network communication, we used the Euclidean distance between region centroids to guide navigation (Seguin et al., 2019; Seguin et al., 2018). Navigating from node *i* to node *j* involves progressing to *i*’s neighbor that is closest in distance to *j*. This process is repeated until *j* is reached (successful navigation) or a node is revisited (failed navigation). Successful navigation path lengths are defined as *Λ*(*i*,*j*) = *L*(*i*,*u*) + … + *L*(*v*,*j*), where {*u*,…,*v*} is the sequence of nodes visited during the navigation from *i* to *j*. Failure to navigate from *i* to *j* yields Λ(i,j)=∞. Navigation efficiency was defined as *NE* = 1/*Λ*. Binary (*NE*_*bin*_), weighted (*NE*_*wei*_), and distance (*NE*_*dis*_) navigation efficiency matrices were computed based on *L*_*bin*_, *L*_*wei*_, *L*_*dis*_, respectively.

#### Diffusion efficiency.

Diffusion efficiency models neural signaling in terms of random walks. Let *T* ∈ℝ^*N*×*N*^ denote the transition matrix of a Markov chain process unfolding on the connection weight matrix *W*. The probability that a naive random walker at node *i* will progress to node *j* is given by $T(i,j)=W(i,j)/\sum_{u=1}^{N}W(i,u)$. The mean first passage time *H*(*i*,*j*) quantifies the expected number of intermediate regions visited in a random walk from *i* to *j* (details on the mathematical derivation of *H* from *T* are given in Fornito et al., 2016; Goñi et al., 2013; Zhou, 2003). Diffusion efficiency is defined as *DE* = 1/*H*, thus capturing the efficiency of neural communication under a diffusive propagation strategy (Goñi et al., 2013). Binary (*DE*_*bin*_), weighted (*DE*_*wei*_), and distance (*DE*_*dis*_) diffusion efficiency matrices were computed based on *W*_*bin*_, *W*_*wei*_, *W*_*dis*_, respectively.

#### Search information.

Search information is derived from the probability of random walkers serendipitously traveling along the shortest paths between node pairs (Rosvall et al., 2005). Let *Ω*(*i*,*j*) = {*u*,…,*v*} be the sequence of nodes along the shortest path from node *i* to node *j* computed from the connection length matrix *L*. The probability that a random walker starting from *i* reaches *j* via *Ω*(*i*,*j*) is given by *P*(*Ω*(*i*,*j*)) = *T*(*i*,*u*) ×… × *T*(*v*,*j*), where *T* is the previously defined transition probability matrix computed from *W*. We defined search information as *SI*(*i*,*j*) = *log*_2_(*P*(*Ω*(*i*,*j*))) (Goñi et al., 2014; Seguin et al., 2019). This definition quantifies how accessible shortest paths are to naive random walkers, capturing the degree to which efficient routes are hidden in network topology. Note that the computation of search information depends on both *L*—for the identification of shortest paths—and *W*—for the simulation of random walks. We used *W*_*wei*_ combined with *L*_*bin*_, *L*_*wei*_, and *L*_*dis*_ to compute, respectively, binary, weighted, and distance versions of search information.

#### Communicability.

Communicability models neural signaling as a diffusive process unfolding simultaneously along all possible walks in a network (Estrada & Hatano, 2008). Communicability between nodes *i* and *j* is defined as the weighted sum of the total number of walks between them, with each walk weighted by its length (i.e., number of connections traversed). In the binary case, this yields $CMY(i,j)=\sum_{n=0}^{\infty }W_{bin}{\left(i,j\right)}^{n}/n!$. In the limit n→∞, this sum converges to $CMY(i,j)=e^{W_{bin}(i,j)}$. Nonbinary connection weight matrices are typically normalized as $W_{wei}^{\prime }(i,j)=W_{wei}(i,j)/(\sqrt{s(i)}\sqrt{\left(s(j)\right)})$ prior to the computation of communicability to attenuate the influence of high strength nodes (Crofts & Higham, 2009), where $s(i)=\sum_{u=1}^{N}W_{wei}(i,u)$ is the total strength of node *i*. We used *W*_*bin*_, *W*_*wei*_*′*, and *W*_*dis*_*′* to compute, respectively, binary, weighted, and distance versions of communicability.

### Functional Connectivity Data

Minimally preprocessed resting-state functional MRI data from the same 889 individuals was also obtained from the HCP. Participants were scanned twice (right-to-left and left-to-right phase encodings) on two separate days, resulting in a total of four sessions per individual. In each session, functional MRI data were acquired for a period of 14 min 33 s with 720 ms TR. (Further details on resting-state functional MRI data collection and preprocessing are described in Glasser et al., 2013; Smith et al., 2013). Functional activity in each of *N* = 68,360 regions was computed by averaging the signal of all vertices comprised in the region. Pairwise Pearson correlation matrices were computed from the regional time series of each session, resulting in four matrices per participant. For each participant, the four matrices were averaged to yield a final *N* × *N* FC matrix. Group-level functional connectomes were computed by averaging the FC matrices of all subjects.

### Behavioral Dimensions

Information on HCP behavioral protocols and procedures is described elsewhere (Barch et al., 2013). A total of 109 variables measuring alertness, cognition, emotion, sensory-motor function, personality, psychiatric symptoms, substance use, and life function were selected from the HCP behavioral dataset (Tian et al., 2020). Selected items consisted of raw (age- and gender-unadjusted), total, or subtotal assessment scores. The set of 109 measures was submitted to an independent component analysis (ICA) pipeline in order to derive latent variables summarizing orthogonal dimensions of behavioral information. This procedure contributed to the computational tractability of our analyses by enabling behavioral inferences to be performed on a small set of data-driven components, rather than being restricted to arbitrarily selected measures.

Behavioral dimensions were computed as follows. A rank-based inverse Gaussian transformation (Van der Waerden, 1953) was used to normalize continuous behavioral variables (87 of 109). Age and gender were regressed out from all behavioral items. ICA was performed on the resulting residuals using the FastICA algorithm (Hyvärinen, 1999) implemented in the *icasso* MATLAB package (Himberg, Hyvärinen, & Esposito, 2004). Participants were sampled with replacement to generate a total of 500 bootstrap samples. ICA was independently performed on each sample with randomly selected initial conditions. Agglomerative clustering with average linkage was used to derive consensus clusters of independent components across different bootstrap samples and initial conditions. This procedure, including bootstrapping and randomization of initial conditions, was repeated for 10 trials of a set of candidate ICA models ranging from 3 to 30 independent components. The best number of components was estimated based on the reproducibility across the 10 trials by means of a cluster quality index. Clearly separated clusters indicate independent components were consistently and reliably estimated, despite being computed based on different bootstrap samples and initial conditions. This criterion identified the five-component model as the most robust and parsimonious set of latent dimensions. This enabled the characterization of the five dimensions as cognitive performance, illicit substance use, tobacco use, personality and emotion traits, and mental health. Further details on the computation of the behavioral dimensions are provided in Tian et al. (2020).

### Behavioral Prediction Framework

Let *y* ∈ℝ^*n*×1^ be a vector of response variables corresponding to a given behavioral dimension, where *n* = 899 is the number of individuals in our sample. Let *X* ∈ℝ^*n*×*p*^ be a matrix of *p* explanatory variables corresponding to the upper triangle of vectorized communication matrices *C* ∈ℝ^*N*×*N*^, so that *p* = *N*(*N* − 1)/2. We applied two independent statistical models to predict *y* from *X*: lasso regression and a regression model based on network features identified by the NBS. These models implement different strategies of feature selection aimed at identifying a parsimonious set of variables in *X* to predict *y*.

The data were split into train and test sets to perform tenfold cross validation. The family structured in the HCP dataset was taken into account by ensuring that individuals of the same family were not separated between train and test sets (Li et al., 2019). Sensitivity to particular train-test data splits was addressed by repeating the tenfold cross validation 10 times. The same train and test sets were used for lasso and NBS regressions. Model parameter estimation was performed exclusively on train sets, while model performance was assessed exclusively on test sets.

#### Lasso regression.

Let {*X*_*a*_,*y*_*a*_} and {*X*_*e*_,*y*_*e*_} denote a split of {*X*,*y*} into train and test sets, respectively. We used lasso regression (Tibshirani, 1996) to compute *β* as

$$\underset{\beta \in R^{p}}{\min}\{\frac{1}{n}∥y_{a}-X_{a}\beta {∥}_{2}^{2}+\lambda ∥\beta {∥}_{1}\},$$

where 0 ≤ *λ* ≤ 1 is a feature selection hyperparameter controlling model complexity. For each outer training set *X*_*a*_, a nested tenfold cross validation was carried out to tune *λ*. This was performed using the MATLAB function *cvglmnet* (Qian, Hastie, Friedman, Tibshirani, & Simon, 2013). For each inner training fold, solutions were computed for a decreasing sequence of *K* logarithmically spaced hyperparameters from *λ*_*max*_ to *λ*_*min*_, where *λ*_*max*_ was the smallest *λ* such that ∥*β*_*inner*_∥_1_ = 0 and *λ*_*min*_ = *ϵλ*_*max*_. Values of *K* and *ϵ* were separately determined for each inner fold by the algorithm, with typical values around *K* = 100 and *ϵ* = 0.01 (further details are described in Friedman, Hastie, & Tibshirani, 2010). The obtained *K* models were then evaluated on the inner test folds. The *λ* resulting in the highest accuracy averaged across inner test folds was selected and used to compute *β* for the outer training set *X*_*a*_. Model fit for each outer fold was evaluated as the Pearson correlation coefficient between $\hat{y_{e}}$ and *y*_*e*_, where $\hat{y_{e}}=X_{e}\beta$. This procedure was repeated for 100 pairs of outer train and test tests (10 repetitions of tenfold splits).

#### Network-based statistics regression.

The NBS identifies sets of connected components in a network that explain significant variation in a response variable (Zalesky et al., 2010). We used the NBS as a feature selection technique to identify behaviorally relevant groups of connections. We then fit a regression model to the average connection weight of the selected connections in order to predict behavior. Importantly, connected components were identified exclusively in training sets, while prediction accuracy was computed based on held-out test sets.

Let {*X*_*a*_,*y*_*a*_} and {*X*_*e*_,*y*_*e*_} denote a split of {*X*,*y*} into train and test sets, respectively. The cross-validated predictive utility of NBS connected components was computed as follows. For each column of *X*_*a*_ (corresponding to the value of a connection in the upper triangle of a communication matrix *C*(*i*,*j*) across subjects in the train set), the interindividual Pearson correlation between *C*(*i*,*j*) and *y*_*a*_ was computed. Connections for which statistical association strength exceeded a *t* statistic threshold *t* > |3| were grouped into sets of positive (*t* > 3) and negative (*t* < −3) connected components. This procedure was repeated for 1,000 random permutations of *y*_*a*_, and the likelihood of observing positive and negative connected components as large as empirical ones was assessed using a nonparametric test. Further details on the NBS are found in Zalesky et al. (2010).

Let *Γ*^+^ and *Γ*^−^ be, respectively, the largest positive and negative connected components identified by the NBS based on the train data {*X*_*a*_,*y*_*a*_}. We computed $g_{a}^{+,-}\in R^{|y_{a}|\times 1}$ as the average weight of connections belonging to the connected component *Γ*^+,−^:

$$g_{a}^{+,-}=\frac{1}{\left|\Gamma^{+,-}\right|}\underset{u\in \Gamma^{+,-}}{\sum }X_{a}(.,u),$$

where |*Γ*^+,−^| indicates the number of connections comprising the connected component. We defined the matrix $G_{a}=[g_{a}^{+}|g_{a}^{-}]$. Therefore, $G_{a}\in R^{|y_{a}|\times 2}$ contains the average weight of connections identified as positively and negatively associated with the behavioral dimension *y* for subjects in the train set {*X*_*a*_,*y*_*a*_}. Using a bivariate linear regression model, we computed the coefficients *β* such that

$$\underset{\beta \in R^{2}}{\min}\{\frac{1}{n}∥y_{a}-G_{a}\beta {∥}_{2}^{2}\}.$$

Analogously, we computed the average weight of connected components in the test set

ge+,−=1|Γ+,−|∑u∈Γ+,−Xe(.,u)

and $G_{e}=[g_{e}^{+}|g_{e}^{-}]$. Finally, behavioral predictions were computed as $\hat{y_{e}}=G_{e}\beta$, and out-of-sample prediction accuracy was evaluated as the Pearson correlation coefficient between $\hat{y_{e}}$ and *y*_*e*_. This procedure was repeated for 100 pairs of train and test sets (10 repetitions of tenfold splits). In cases where no significant component was identified by the NBS (|*Γ*^+,−^| = 0), model performance was set to 0.

### Functional Connectivity Prediction Framework

FC predictions were computed as the Spearman correlation between empirical FC and analytically derived communication matrices. None of the network communication models and measures used to infer FC required training, statistical estimation of weights, or parameter tuning (an advantage over other classes of high-order models). Hence, we oftentimes adopted the term FC “prediction” even though predictive utility was not assessed out of sample (Goñi et al., 2014).

## ACKNOWLEDGMENT

We thank Olaf Sporns for valuable discussions. Human data were provided by the Human Connectome Project, WUMinn Consortium (1U54MH091657; principal investigators: David Van Essen and Kamil Ugurbil) funded by the 16 National Institutes of Health (NIH) institutes and centers that support the NIH Blueprint for Neuroscience Research; and by the McDonnell Center for Systems Neuroscience at Washington University.

## SUPPORTING INFORMATION

Supporting information for this article is available at https://doi.org/10.1162/netn_a_00161.

## AUTHOR CONTRIBUTIONS

Caio Seguin: Conceptualization; Data curation; Formal analysis; Methodology; Visualization; Writing – original draft; Writing – review & editing. Ye Tian: Data curation; Formal analysis; Methodology; Writing – original draft. Andrew Zalesky: Conceptualization; Formal analysis; Methodology; Writing – original draft; Writing – review & editing.

## FUNDING INFORMATION

Caio Seguin, Melbourne Research Scholarship, University of Melbourne (http://dx.doi.org/10.13039/501100000987). Andrew Zalesky, National Health and Medical Research Council (AU), Award ID: 1136649.

## TECHNICAL TERMS

Polysynaptic path: A path between a source region and target region that contains at least one intermediary region; a path between anatomically unconnected regions.
Brain network communication model: A model of how neural signaling unfolds atop the structural connectome; a strategy to describe how information is communicated between regions.
Structure-function coupling: Relationship between structural and functional connectivity. Typically quantified by the correlation between structural and functional connection weights.
Shortest paths efficiency: Communication efficiency under the shortest path routing model. Considers that neural signaling unfolds via optimally efficient paths.
Navigation efficiency: Communication efficiency under the navigation model. Considers that neural signaling unfolds via geometrically greedy paths.
Diffusion efficiency: Communication efficiency under the diffusion model. Considers that neural signaling unfolds via random walks.
Search information: The amount of information required for a random walker to travel via shortest paths. Quantifies the accessibility of efficient communication paths in the connectome.
Communicability: Weighted sum of all walk lengths between region pairs. Considers that neural signaling unfolds as a diffusive broadcasting process.
Communication matrix: A matrix quantifying communication between every region pair under the assumption of a certain network communication model.
