# Supplementary material for: Network communication models improve the behavioral and functional predictive utility of the human structural connectome
Source: Netw Neurosci. 2020 Nov 1;4(4):980–1006. doi: 10.1162/netn_a_00161 (PMC7655041; doi:10.1162/netn_a_00161)
Supplement: Supplementary file 1 [file netn-04-980-s001.pdf]

Seguin, C., Tian, Y. & Zalesky, A. (2020). Supporting information for "Network communication models improve the behavioral and functional predictive utility of the human structural connectome." *Network Neuroscience*, *4*(4), 980-1006. https://doi.org/10.1162/netn_a_00161

Supplementary Material to Network communication models improve the behavioral and functional predictive utility of the human structural connectome

Caio Seguin[1],* Ye Tian[1], and Andrew Zalesky[1,2]
[1] *Melbourne Neuropsychiatry Centre, The University of Melbourne and Melbourne Health, Melbourne, VIC 3010, Australia and*
[2] *Department of Biomedical Engineering, Melbourne School of Engineering, The University of Melbourne, Melbourne, VIC 3010, Australia*
(Dated: July 30, 2020)

SUPPLEMENTARY MATERIAL

Note 1: Null models of behavioral predictions

We sought to compare the behavioral predictions reported in Fig. 3 (Lasso regression, $N = 360$ thresholded connectomes) to predictions obtained for communication models computed on top of randomized structural connectomes. Structural connectomes were rewired using the Maslov Sneppen routine [1], which randomizes network topology while preserving connection density and degree distribution. Null estimates of behavioral prediction accuracy were computed as follows. The structural connectome of each subject was independently rewired resulting on one null dataset containing 889 randomized SC matrices. We then followed the same procedure used to predict behavior from empirical SC (Fig. 1). Null communication matrices were computed from randomized connectomes for all 15 network communication models, amounting to a total of 16 null datasets (FC was not considered in this analysis). Using lasso regression, the null datasets were used to derive out-of-sample predictions of the behavioral dimensions for 10 repetitions of 10-fold cross validation. This resulted in 100 null prediction accuracy estimates for each of the 16 predictors. Lastly, to account for within subject variations in the rewiring routine, the entire null behavioral prediction process was repeated 5 times (i.e., for 5 different initial sets of randomized SC matrices). Final null prediction accuracies were averaged across the 5 repetitions. As with empirical results, we considered the average null prediction accuracy obtained for cognition and tobacco use components.

Figure S4a shows the empirical prediction accuracy distributions paired with their null counterparts. All communication models showed significantly greater average prediction accuracy when computed on empirical connectomes rather than randomized ones (Fig. S4b). The sole exception was the binary diffusion model, which was the worst-ranking model when computed on empirical connectomes. In fact, we observed that models with higher empirical predictive utility tended to outperform their null counterparts by larger margins (Spearman rank correlation $r_{(15)} = 0.89$, $p = 0$ between empirical accuracy and the t-statistics of pairwise comparisons of models to their null counterparts). In addition, when comparing accuracies across all possible pairs of empirical and null predictors, we found that most empirical models significantly outperformed most null models. This is evidenced by the predominantly warm- and cool-colored off-diagonal blocks in the effect size matrix shown in Figure S4c.

Together, these findings provide further evidence that network communication models are capable of predicting interindividual variation in human behavior, and that observed differences in empirical accuracies reflect meaningful distinctions in the predictive utility of different models. Disruptions to SC topology, even when preserving degree distribution, led to significant reductions in the predictive utility of communication models. This suggests that the relationship between network communication models and behavior is contingent on high-order organizational features of the human structural connectome.

Note 2: Impact of cross validation repetitions on behavioral predictive utility comparisons

The behavioral predictive utility analyses performed in this paper were based on distributions comprising 100 estimates of prediction accuracy, computed by performing 10 repetitions of 10-fold cross validation. In this section, we evaluate whether our results are stable when considering single repetitions of the 10-fold cross validation. In other words, we aimed to compare communication models based on distributions comprising 10 estimates of prediction accuracy, one from each cross validation fold. Our goal was to determine whether statistical differences between predictors (or the lack thereof) reported when considering 100 accuracy estimates would be observed when only taking into account the accuracies from each individual cross validation repetition.

To this end, we separately recomputed the results shown in Fig. 3 for each of the 10 repetitions of the cross validation process. We derived 10 rankings of predictors (as in Fig. 3a) and 10 17×17 effect size matrices of pairwise statistical comparisons (as in Fig. 3b). We also computed a ranking of predictors and effect size matrix for the accuracy distributions obtained when averaging results across the cross validation repetitions. This resulted in a set of 11 predictor rankings and effect size

* caioseguin@gmail.com

matrices.

To facilitate comparisons, effect size matrices were binarized. Entries equal to 1 and 0 denoted, respectively, evidence for significant and non-significant differences between accuracy distributions (Bonferroni-corrected threshold for multiple comparisons $\alpha = 0.05/(17 \times 16/2) = 3.67 \times 10^{-4}$). This categorization of statistical test outcomes allowed us to compare our original results to the ones from individual cross validation repetitions by means of Fisher's exact tests. Rejection of the null hypothesis provides evidence of nonrandom associations between significance outcomes computed on 100 accuracy estimates to those based on 10 accuracy estimates.

Results are shown in Table S1. We found that the average prediction accuracies shown in Fig. 3a were strongly correlated to those obtained for individual repetitions of the cross validation process. In addition, for each individual repetition, the outcome of pairwise statistical comparisons between predictors was significantly associated to the original outcomes shown in Fig. 3b.

Note 3: Behavioral predictive utility for the cognition and tobacco use dimensions

We have characterized the predictive utility of connectivity measures and communication models by considering a pooled prediction accuracy between the cognition and tobacco use dimensions. As we have seen, these behavioral dimensions led to the most accurate and consistent predictions in our sample. Considering the average prediction accuracy across the two traits facilitated the comparison of communication models by providing us with a single score on which to evaluate prediction accuracy. However, this approach may potentially obscure nuanced phenotype-specific relationships between brain and behavior. Indeed, we observed no correlation between the prediction accuracies obtained for cognition and tobacco use (Spearman rank correlation $p = 0.49, 0.69$ for lasso and NBS, respectively). Therefore, in this section, we sought to separately examine behavioral predictions for the cognition and tobacco use dimensions.

We observed that the outstanding performance of FC was mostly due to the cognition dimension (Fig. S5 and S8; this can also be observed in Fig. 2c,d). This is in line with several findings on the relationship between cognitive processes and the architecture of functional networks [2]. Interestingly, while FC still yielded top-ranking predictions of tobacco use, several communication models showed comparable predictive utility in this dimension (Fig. S6 and S9; again evident in Fig. 2c,d). For instance, navigation was the best predictor of tobacco use, with binary and distance navigation occupying the first positions under lasso and NBS, respectively. Although none of the communication models statistically outperform FC, this points towards a tighter margin of difference between the utility of structural and functional measures in predicting behavioral phenotypes not directly related to cognition.

Another interesting observation was that weighted search information was the best communication model in predicting cognition, but showed near bottom-ranking predictive utility of tobacco use, which resulted in a moderate performance when combining predictions from both behavioral dimensions. Along similar lines, we observed that rankings of connection weight definitions were different across behavioral dimensions and prediction methods, painting an unclear picture of what weighting schemes best contribute to predict human behavior from structural connectomes.

Therefore, collectively, our results did not point towards a single communication model as the best predictor of human behavior. Despite the overall good performance of communicability and navigation, our observations indicate that different communication models may be better suited to predict different behavioral dimensions, possibly suggesting the presence of behavior-specific signaling mechanisms in the human brain. Importantly, across the multiple analyses performed, our results consistently suggested that network communication models, in particular communicability and navigation, improve the behavioral predictive utility of the human connectome.

Note 4: Additional analyses of structure-function coupling

We further examined structure-function relationships by stratifying FC predictions across anatomically connected and unconnected regions pairs (Fig. S11a). In accordance to previous work [3, 4], associations to FC were stronger for connected regions. Despite these changes in association strength, rankings of communication models in terms of FC predictions were consistent across the scenarios explored (Spearman rank correlation $r = 0.90, 0.68, 0.85$ and $p = 8 \times 10_{-7}, 3 \times 10_{-3}, 0$ between connected and all, all and unconnected, and connected and unconnected region pairs, respectively). Interestingly, certain communication models outperformed SC for connected regions, suggesting that indirected polysynaptic signaling may be relevant for communication even in the presence of direct anatomical links.

Estimates of structure-function coupling depend on accurate reconstruction of structural connectomes. However, white matter tractography is prone to a number of known biases, of which the underestimation of interhemispheric connections is an important concern [5]. To attenuate this issue, past studies have focused on intrahemispheric characterization of structure-function coupling [6, 7]. Focusing on the right hemisphere, we found that, for all communication models, FC associations were stronger compared to whole-brain estimates (Fig. S11b). Importantly, the functional predictive utility ranking of communication models remained consistent (Spearman rank correlation $r = 0.93$ and $p = 0$ between whole-brain

and right hemisphere rankings). This suggests that our analyses may provide a meaningful ranking of signaling strategies despite shortcomings in connectome mapping techniques.

[1] S. Maslov and K. Sneppen, “Specificity and stability in topology of protein networks,” *Science*, vol. 296, pp. 910–3, May 2002.

[2] J. D. Medaglia, M.-E. Lynall, and D. S. Bassett, “Cognitive network neuroscience,” *J Cogn Neurosci*, vol. 27, pp. 1471–91, Aug 2015.

[3] J. Goñi, M. P. van den Heuvel, A. Avena-Koenigsberger, N. Velez de Mendizabal, R. F. Betzel, A. Griffa, P. Hagmann, B. Corominas-Murtra, J.-P. Thiran, and O. Sporns, “Resting-brain functional connectivity predicted by analytic measures of network communication,” *Proc Natl Acad Sci U S A*, vol. 111, pp. 833–8, Jan 2014.

[4] C. Seguin, M. P. van den Heuvel, and A. Zalesky, “Navigation of brain networks,” *Proc Natl Acad Sci U S A*, vol. 115, pp. 6297–6302, 06 2018.

[5] K. H. Maier-Hein, P. F. Neher, J.-C. Houde, M.-A. Côté, E. Garyfallidis, J. Zhong, M. Chamberland, F.-C. Yeh, Y.-C. Lin, Q. Ji, W. E. Reddick, J. O. Glass, D. Q. Chen, Y. Feng, C. Gao, Y. Wu, J. Ma, H. Renjie, Q. Li, C.-F. Westin, S. Deslauriers-Gauthier, J. O. O. González, M. Paquette, S. St-Jean, G. Girard, F. Rheault, J. Sidhu, C. M. W. Tax, F. Guo, H. Y. Mesri, S. Dávid, M. Froeling, A. M. Heemskerk, A. Leemans, A. Boré, B. Pinsard, C. Bedetti, M. Desrosiers, S. Brambati, J. Doyon, A. Sarica, R. Vasta, A. Cerasa, A. Quattrone, J. Yeatman, A. R. Khan, W. Hodges, S. Alexander, D. Romascano, M. Barakovic, A. Auría, O. Esteban, A. Lemkaddem, J.-P. Thiran, H. E. Cetingul, B. L. Odry, B. Mailhe, M. S. Nadar, F. Pizzagalli, G. Prasad, J. E. Villalon-Reina, J. Galvis, P. M. Thompson, F. D. S. Requejo, P. L. Laguna, L. M. Lacerda, R. Barrett, F. Dell'Acqua, M. Catani, L. Petit, E. Caruyer, A. Daducci, T. B. Dyrby, T. Holland-Letz, C. C. Hilgetag, B. Stieltjes, and M. Descoteaux, “The challenge of mapping the human connectome based on diffusion tractography,” *Nat Commun*, vol. 8, p. 1349, Nov 2017.

[6] P. E. Vértes, A. F. Alexander-Bloch, N. Gogtay, J. N. Giedd, J. L. Rapoport, and E. T. Bullmore, “Simple models of human brain functional networks,” *Proc Natl Acad Sci U S A*, vol. 109, pp. 5868–73, Apr 2012.

[7] A. Messé, D. Rudrauf, H. Benali, and G. Marrelec, “Relating structure and function in the human brain: relative contributions of anatomy, stationary dynamics, and nonstationarities,” *PLoS Comput Biol*, vol. 10, p. e1003530, Mar 2014.

[8] A. Zalesky, A. Fornito, and E. T. Bullmore, “Network-based statistic: identifying differences in brain networks,” *Neuroimage*, vol. 53, pp. 1197–207, Dec 2010.

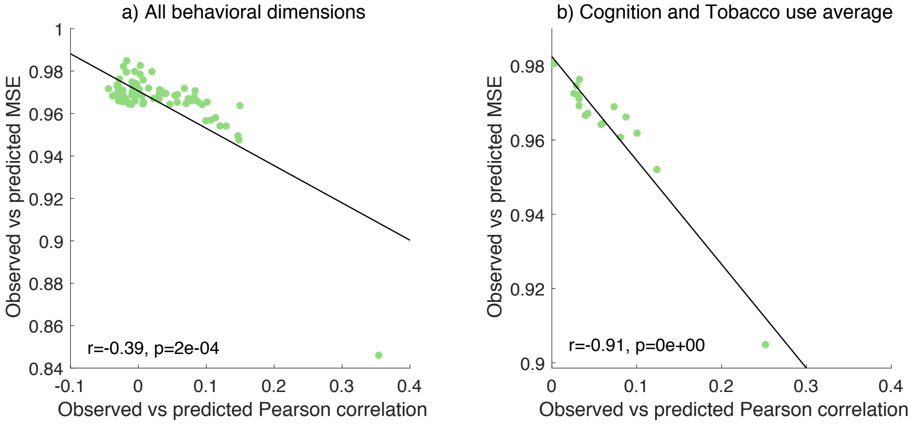

FIG. S1. Comparison between behavioral prediction accuracies computed using Pearson correlation and mean square error evaluated using Spearman rank correlation (Lasso regression, $N = 360$ thresholded connectomes). The least squares line is shown in black.

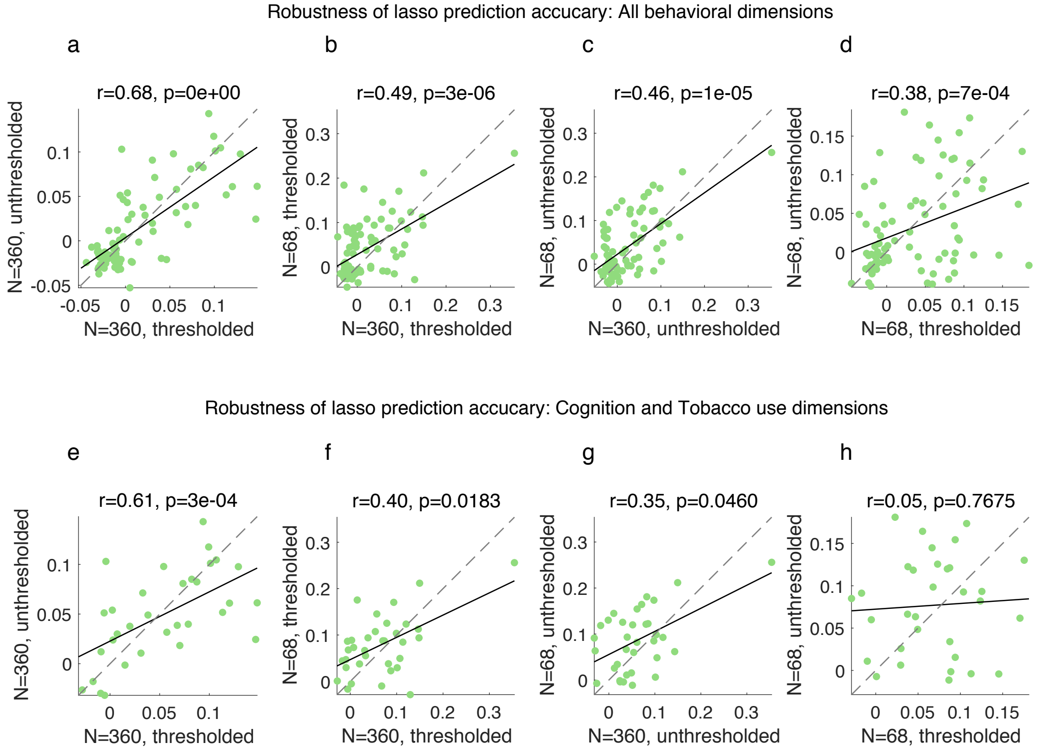

FIG. S2. Comparison between behavioral prediction accuracies across multiple connectome mapping pipelines (Lasso regression). Scatter plots show the least squares and $x = y$ lines are shown in solid black and gray dashed traces, as well as the Spearman rank correlation coefficient and p-value. Comparisons between results obtained for thresholded and unthresholded structural connectomes (panels **a**, **d**, **e**, **h**) do not include prediction accuracies obtained from FC, since functional matrices did not undergo connection thresholding. Comparisons between results obtained for structural connectomes comprising 68 and 360 regions (panels **b**, **c**, **f**, **g**) include prediction accuracies obtained from FC, since functional matrices are influenced by parcellation choice. Removing data points pertaining to FC prediction accuracies from panels **b**,**c**,**f**,**g** led to Spearman rank correlation $r = 0.49, 0.46, 0.29, 0.21$ and $p = 6 \times 10^{-6}, 2 \times 10^{-5}, 0.11, 0.24$, respectively.

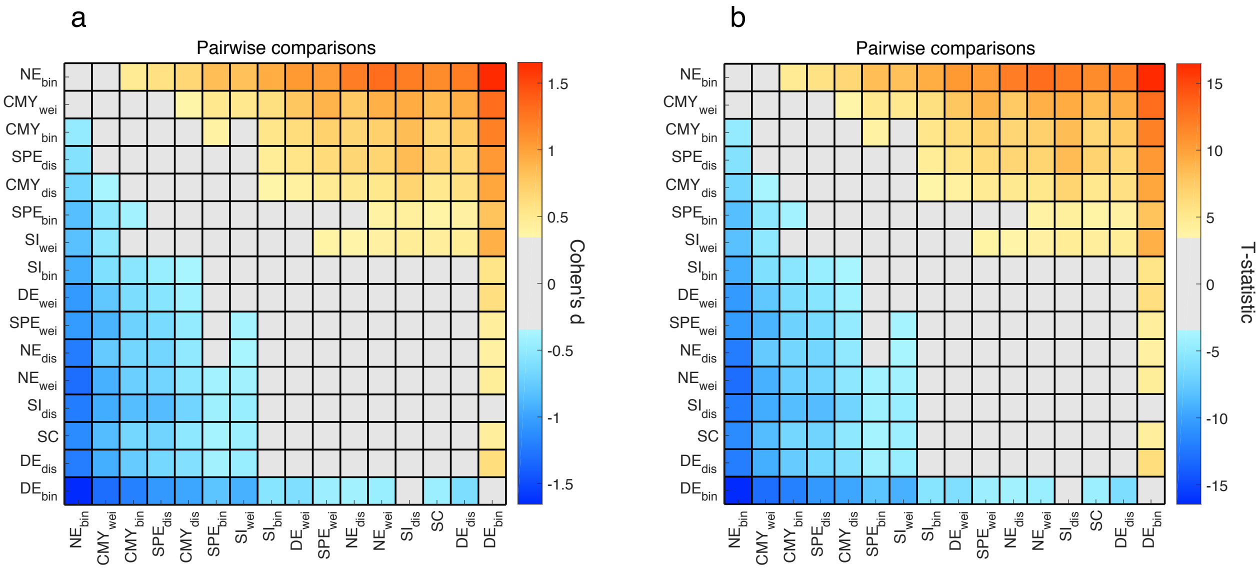

FIG. S3. Effect size matrices showing **(a)** Cohen's d and **(b)** t-statistics from pairwise statistical comparisons between network communication models and SC (same as Fig. 3b but excluding comparisons to FC for better contrast in effect size values; Lasso regression, $N = 360$ thresholded connectomes, average cognition and tobacco use prediction accuracies).

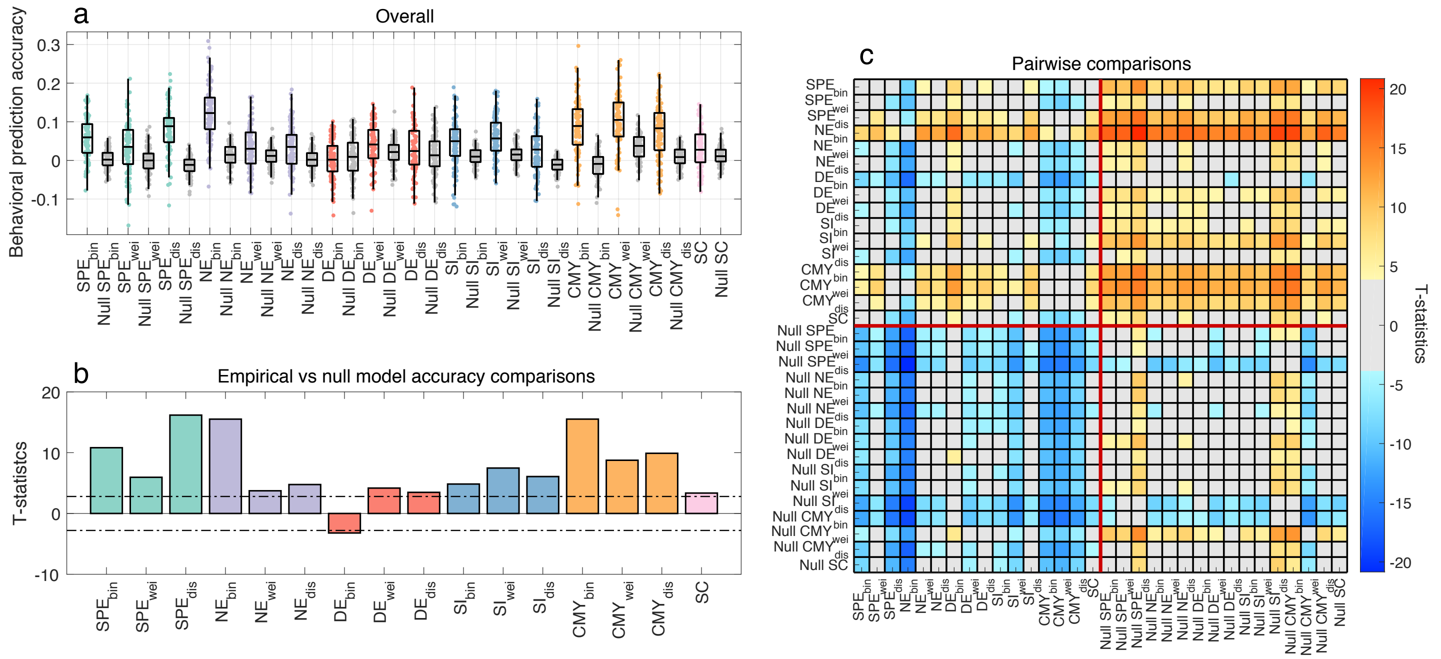

FIG. S4. Comparison of the behavioral predictive utility of network communication models computed on empirical and randomized connectomes (NBS, $N = 360$ thresholded connectomes, average cognition and tobacco use prediction accuracies). **(a)** Boxplots indicate the prediction accuracy distributions for 10 repetitions of 10-fold cross-validation. Boxplots are ordered to contrast empirical accuracy distributions (colored) with their null counterparts (grey). **(b)** T-statistics obtained from pairwise repeated-measure t-tests between empirical prediction accuracies and their null counterparts. Positive statistics indicate that models computed on empirical connectomes outperformed those computed on randomized connectomes. The dotted line denotes the Bonferroni-corrected significance threshold for 16 multiple comparisons ($\alpha = 0.0031$). **(c)** Effect size matrix of pairwise repeated-measures t-tests between distributions in panel (a), with colored cells indicating significant differences in mean prediction accuracies (Bonferroni-corrected for $32 \times 31/2$ multiple comparisons with significance threshold $\alpha = 1 \times 10^{-4}$).

	Comparison to results obtained for 10 repetitions of 10-fold cross validation	
10-fold repetition	Average prediction accuracy correlation (Spearman rank coefficient $r_{(15)}$)	Outcomes from pairwise comparisons between predictors (Fisher's exact test p-value)
#1	0.91	0.0002
#2	0.96	5×10^{-7}
#3	0.96	2×10^{-5}
#4	0.97	0.0023
#5	0.92	2×10^{-5}
#6	0.98	0.0021
#7	0.91	0.0006
#8	0.92	0.0002
#9	0.98	0.0002
#10	0.91	0.0003
Average across repetitions	0.98	5×10^{-19}

TABLE S1. Comparison of results for individual repetitions of 10-fold cross validation (Lasso regression, $N = 360$ thresholded connectomes, average cognition and tobacco use prediction accuracies). For each cross validation repetition, the average prediction accuracy of 16 predictors as well as the outcomes of pairwise statistical tests between predictors are compared to those obtained when pooling prediction accuracies across the 10 repetitions of the cross validation process.

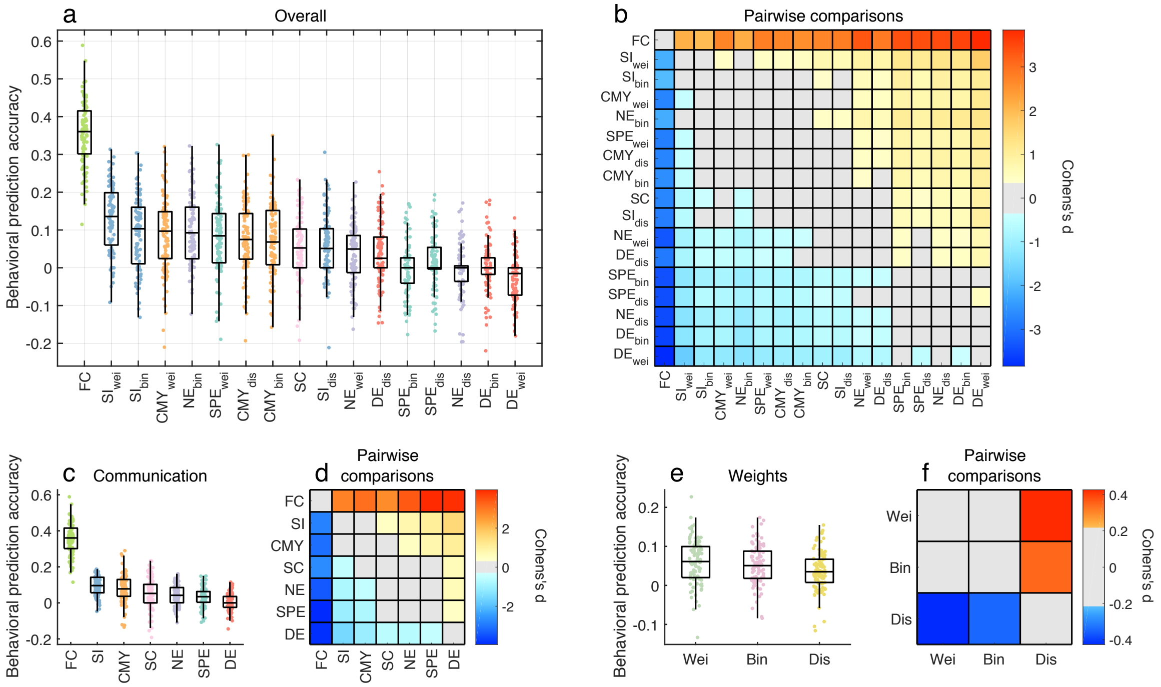

FIG. S5. Comparison of the behavioral predictive utility of connectome communication models (Lasso regression, $N = 360$ thresholded connectomes, cognition prediction accuracy).

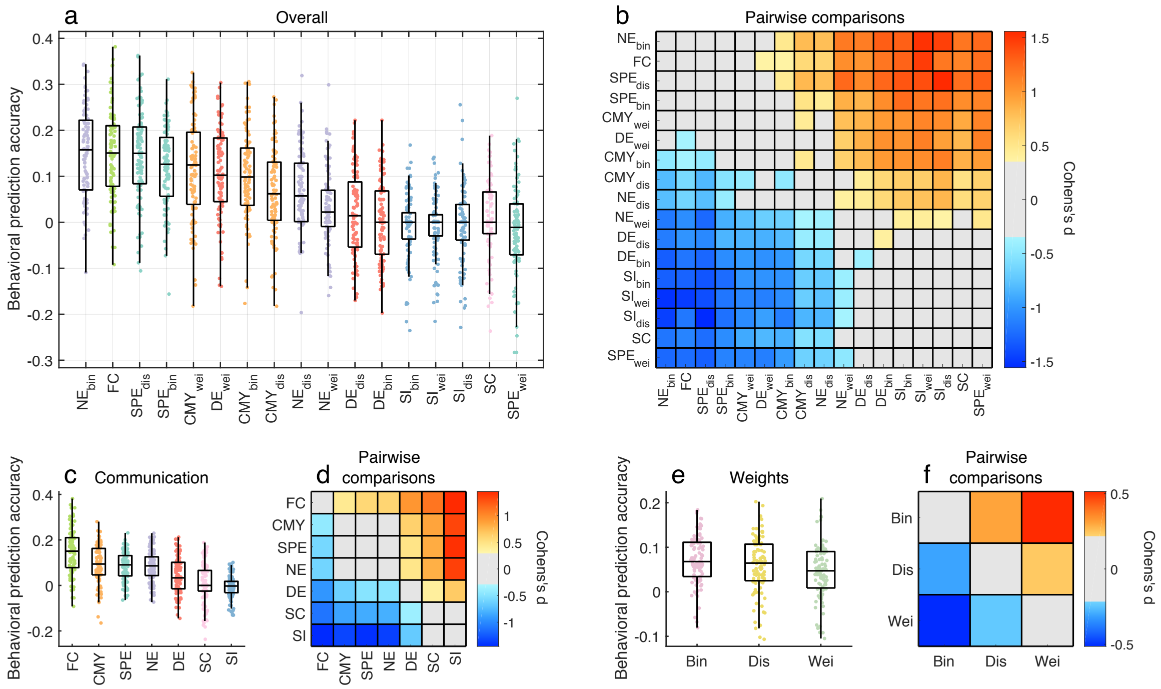

FIG. S6. Comparison of the behavioral predictive utility of connectome communication models (Lasso regression, $N = 360$ thresholded connectomes, tobacco use prediction accuracy).

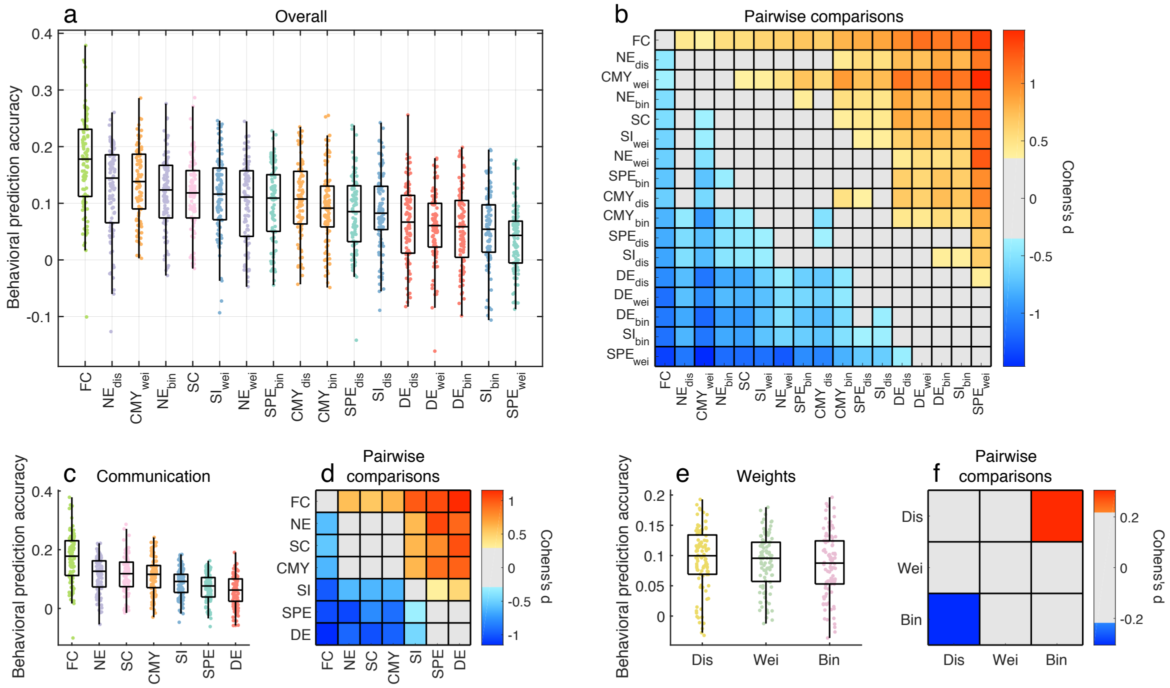

FIG. S7. Comparison of the behavioral predictive utility of connectome communication models (NBS, $N = 360$ thresholded connectomes, average cognition and tobacco use prediction accuracies). It is worth noting that the NBS feature selection process is better suited to sparse graphs [8], which could confer an advantage to sparse SC matrices over fully-connected communication and FC matrices.

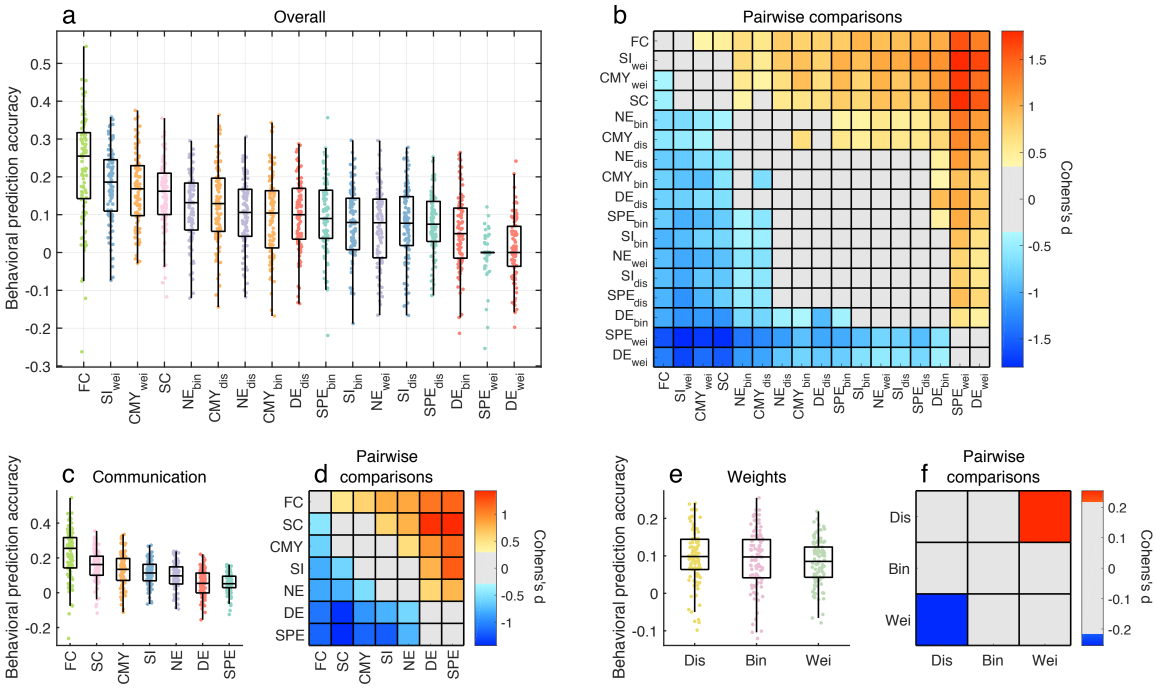

FIG. S8. Comparison of the behavioral predictive utility of connectome communication models (NBS, $N = 360$ thresholded connectomes, cognition prediction accuracy).

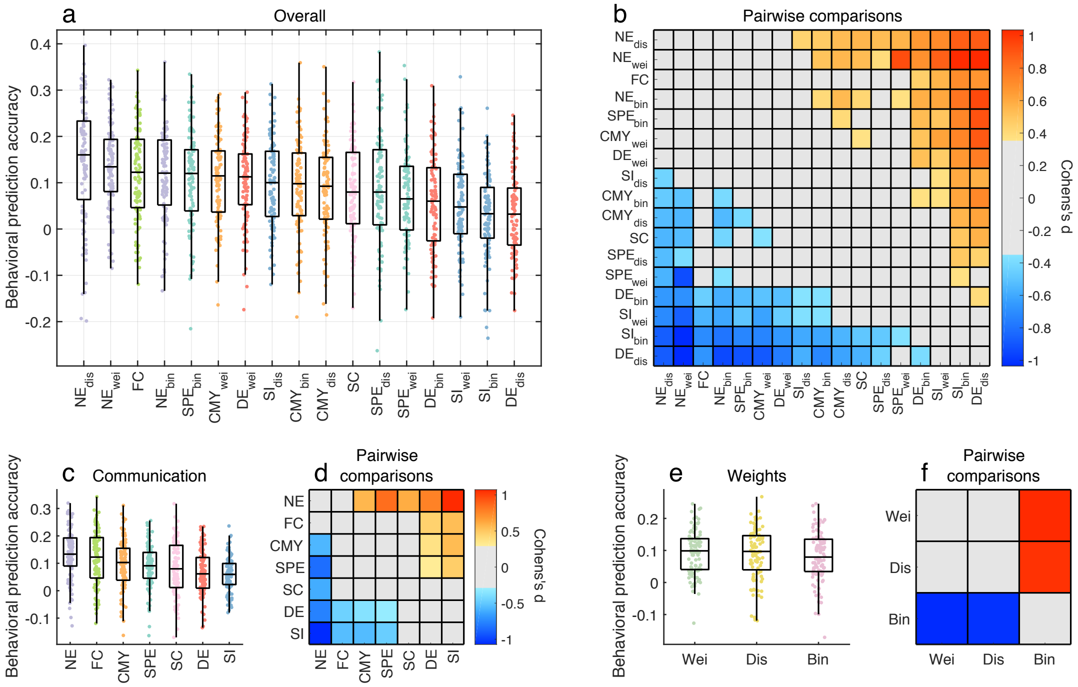

FIG. S9. Comparison of the behavioral predictive utility of connectome communication models (NBS, $N = 360$ thresholded connectomes, tobacco use prediction accuracy).

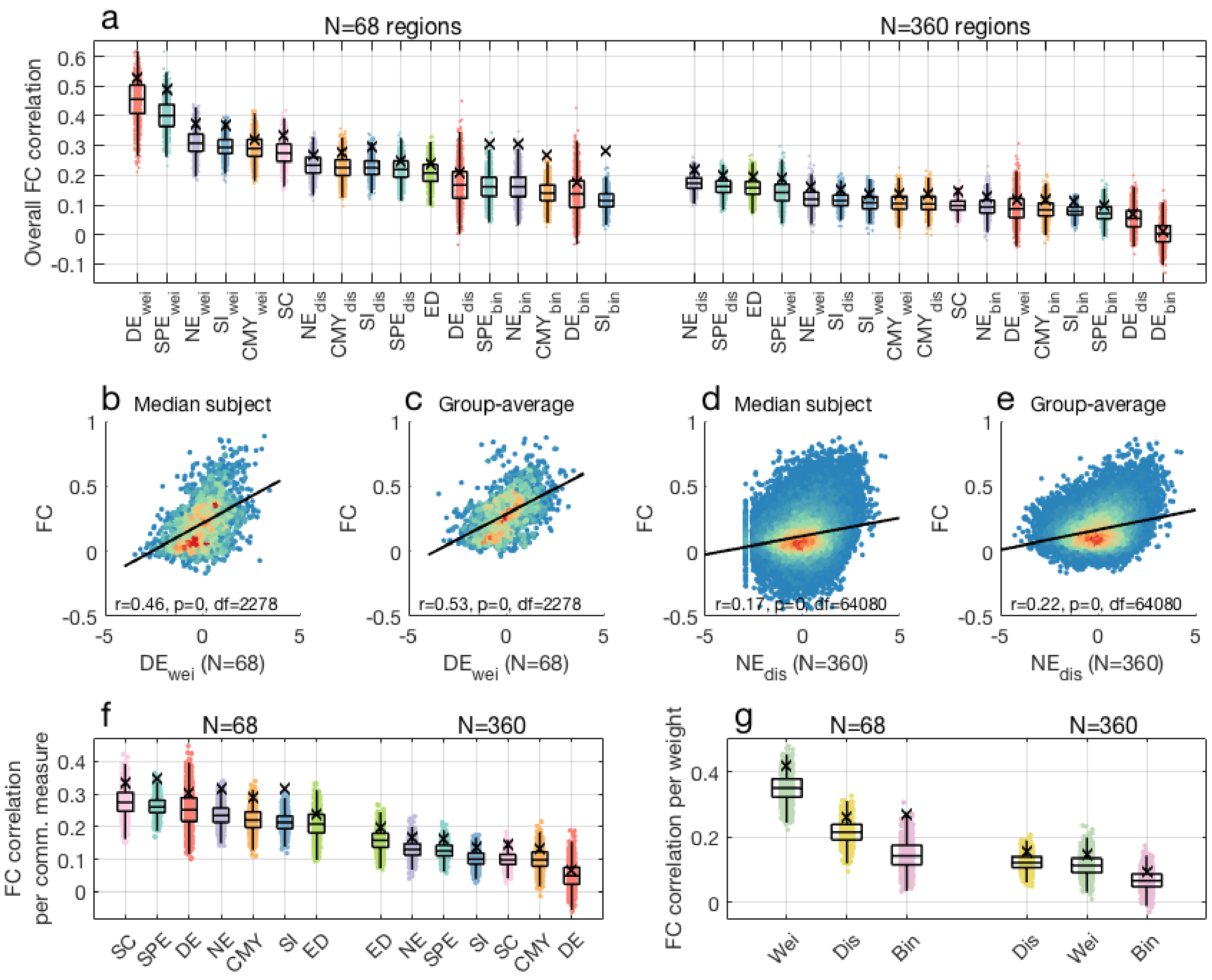

FIG. S10. Structure-function coupling across connectome communication models ($N = 68, 360$ unthresholded connectomes).

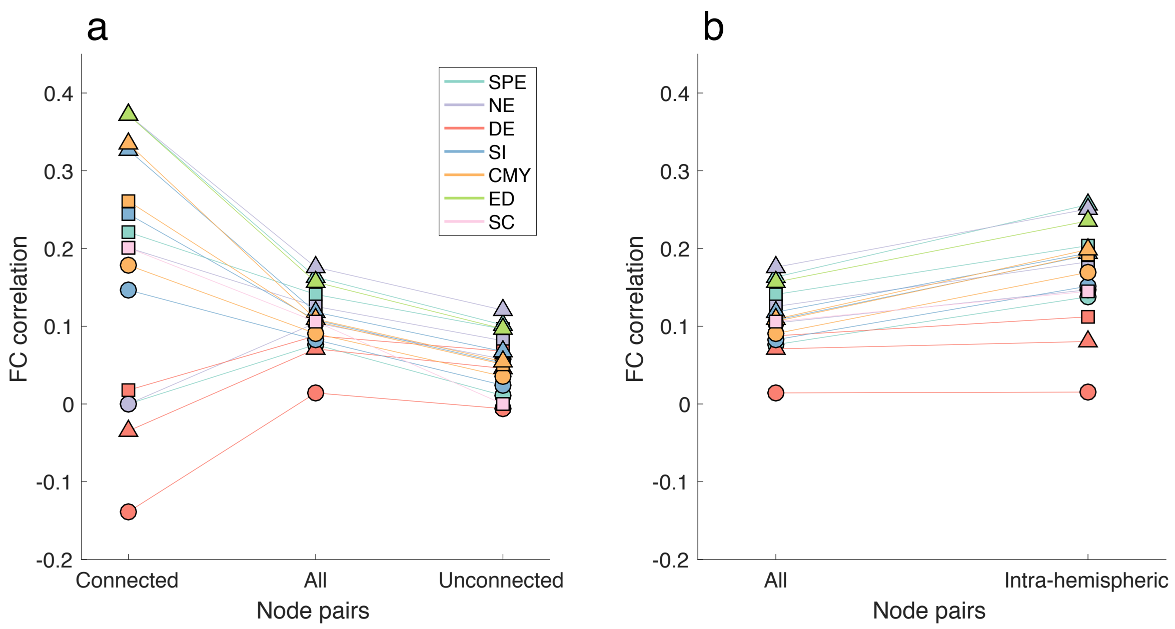

FIG. S11. Stratification of structure-function coupling across connectome communication models ($N = 360$ thresholded connectomes). Markers indicate median (across subjects) Spearman correlation between FC and communication models. Squared, circular and triangular markers denote models computed on weighted, binary and distance connectomes, respectively. Marker colors denote different FC predictors. Teal: shortest paths, violet: navigation, red: diffusion, blue: search information, orange: communicability, pink: SC, and green: Euclidean distance. **(a)** Structure-function coupling anatomically connected (left), all (center) and anatomically unconnected (right) region pairs. **(b)** Structure-function coupling for whole-brain (left) and right-hemisphere (right) connectomes.
